# Cell envelope growth of Gram‐negative bacteria proceeds independently of cell wall synthesis

**DOI:** 10.15252/embj.2022112168

**Published:** 2023-06-01

**Authors:** Enno R Oldewurtel, Yuki Kitahara, Baptiste Cordier, Richard Wheeler, Gizem Özbaykal, Elisa Brambilla, Ivo Gomperts Boneca, Lars D Renner, Sven van Teeffelen

**Affiliations:** ^1^ Morphogenesis and Microbial Growth Lab Institut Pasteur Paris France; ^2^ Université de Paris Paris France; ^3^ Département de Microbiologie, Infectiologie, et Immunologie, Faculté de Médecine Université de Montréal Montréal QC Canada; ^4^ Institut Pasteur, Université Paris Cité, UMR6047 CNRS, U1306 INSERM, Unité Biologie et Génétique de la Paroi Bactérienne Paris France; ^5^ Leibniz Institute of Polymer Research and the Max Bergmann Center of Biomaterials Dresden Germany; ^6^ Present address: Department of Infection Biology London School of Hygiene and Tropical Medicine London UK

**Keywords:** cell‐size growth, Gram‐negative bacteria, mechanical envelope forces, peptidoglycan endopeptidases, peptidoglycan synthesis, Microbiology, Virology & Host Pathogen Interaction

## Abstract

All bacterial cells must expand their envelopes during growth. The main load‐bearing and shape‐determining component of the bacterial envelope is the peptidoglycan cell wall. Bacterial envelope growth and shape changes are often thought to be controlled through enzymatic cell wall insertion. We investigated the role of cell wall insertion for cell shape changes during cell elongation in Gram‐negative bacteria. We found that both global and local rates of envelope growth of *Escherichia coli* remain nearly unperturbed upon arrest of cell wall insertion—up to the point of sudden cell lysis. Specifically, cells continue to expand their surface areas in proportion to biomass growth rate, even if the rate of mass growth changes. Other Gram‐negative bacteria behave similarly. Furthermore, cells plastically change cell shape in response to differential mechanical forces. Overall, we conclude that cell wall‐cleaving enzymes can control envelope growth independently of synthesis. Accordingly, the strong overexpression of an endopeptidase leads to transiently accelerated bacterial cell elongation. Our study demonstrates that biomass growth and envelope forces can guide cell envelope expansion through mechanisms that are independent of cell wall insertion.

## Graphical abstract

Cell wall insertion in *E. coli* and other Gram‐negative rod‐shaped bacteria is dispensable for the cell surface increase upon mass growth and for the cell shape response to mechanical forces.

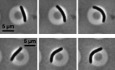

## Introduction

Cell shape and volume of most bacteria are physically determined by the peptidoglycan cell wall. This covalent meshwork surrounds the cell, maintains cell shape, and therefore defines cell volume (Höltje, 1998). During growth, bacteria need to enzymatically cut the cell wall and insert new peptidoglycan material while controlling cell shape (Typas *et al*, 2012; Garde *et al*, 2021; Rohs & Bernhardt, 2021). In Gram‐negative bacteria, such as *Escherichia coli*, the cell wall is a thin layer with a thickness of a few nanometers (Yao *et al*, 1999; Gan *et al*, 2008), which is sandwiched between the two other envelope components, the cytoplasmic membrane and the outer membrane. To prevent cell lysis, cell wall expansion via lytic autolysins and insertion via peptidoglycan synthases are likely coordinated. Accordingly, cell wall insertion might be rate‐limiting for the global rate of cell envelope growth (Höltje, 1998; Vollmer & Bertsche, 2008; Typas *et al*, 2012; Harris & Theriot, 2016), that is the global increase of cell‐surface area, and for the local rates of cell envelope growth, notably during bending or straightening (Cabeen *et al*, 2009; Ursell *et al*, 2014; Wong *et al*, 2017), when a cell expands its envelope faster on one side of the cell than on the other. However, the requirement of cell wall insertion for cell envelope growth has never been strictly demonstrated. Instead, it is possible that cell wall‐cleaving autolysins expand the cell wall independently of cell wall insertion (Singh *et al*, 2012).

This hypothesis is supported by our previous finding that the global rate of cell‐surface growth of *E. coli* is nearly unperturbed upon treatment with the small molecule A22 (Oldewurtel *et al*, 2021). A22 inhibits MreB polymerization and thus the activity of the cell wall‐inserting rod complex (Uehara & Park, 2008). Furthermore, Miguel De Pedro's lab previously reported that *E. coli* continues to grow normally even if cell wall insertion is reduced, using a peptidoglycan‐precursor auxotroph (Prats & De Pedro, 1989) and studying wild‐type cells outgrowing from stationary phase (Pisabarro *et al*, 1987). Thus, the global rates of envelope growth and cell wall insertion can be partially decoupled. Finally, the heterologous expression of two *Vibrio cholerae* proteins (CrvA and CrvB) introduces curvature in multiple Gram‐negative rods independently of both major cell wall synthesis complexes (Martin *et al*, 2021). Some of us previously demonstrated that differential growth is likely coupled to differential areal cell envelope strain, that is, differential local mechanical stretching of the cell wall (Wong *et al*, 2017). It is thus conceivable that mechanical strain directly affects cell wall‐cleaving autolysins, without the need for cell wall‐inserting enzymes.

One important aspect of cell envelope growth is the regulation of cell width. It is well known that MreB‐actin‐based cell wall insertion is required to control cell width in many rod‐shaped bacteria (Typas *et al*, 2012). MreB‐actin orients peptidoglycan insertion (Hussain *et al*, 2018) and possibly exerts a force on the cytoplasmic membrane (Wang & Wingreen, 2013; Hussain *et al*, 2018). However, we recently demonstrated that cell width is equally affected by cytoplasmic osmotic pressure (Oldewurtel *et al*, 2021), possibly through its effect on mechanical cell envelope strain or stress. We hypothesize that pressure‐dependent cell widening might be independent of cell wall insertion.

Here, we demonstrate that selected Gram‐negative rod‐shaped bacteria control both their global rate of cell envelope growth, their local rate of cell bending/straightening, and their pressure‐dependent rate of cell widening independently of cell wall insertion. While cell wall insertion remains essential for cell‐width regulation, cell division, and cell wall integrity, cell wall‐cleaving autolysins control envelope growth independently of peptidoglycan insertion. Accordingly, strong overexpression of a DD‐endopeptidase (MepS) leads to a transient increase in cell envelope growth. However, the increase in surface growth is only of short duration consistent with the control of lytic activity at the enzyme level (Shin *et al*, 2020).

## Results

### The global rate of cell envelope growth is controlled independently of cell wall insertion

**Figure 1:**
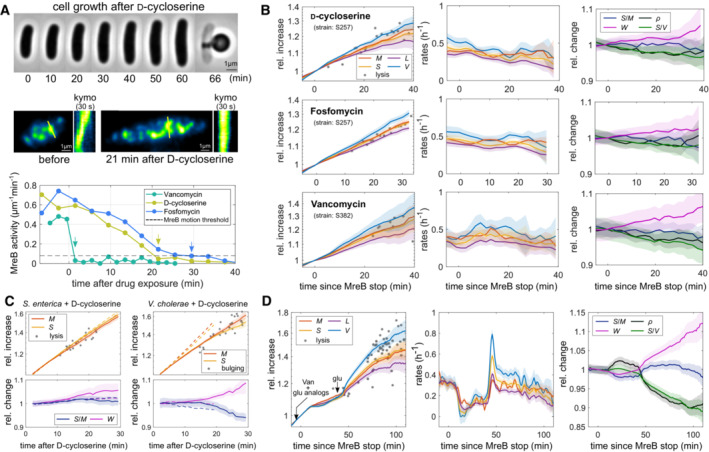
The global rate of surface growth is independent of cell wall synthesis Single‐cell snapshots (top), MreB kymographs (middle), and MreB activity (bottom) of nondividing cells after treatment with cell wall synthesis inhibitors. Cells were treated with 1 mM D‐cycloserine, 500 μg/ml fosfomycin (strain S257, *mreB::mreB‐msfGFP*/pDB192), or 100 μg/ml vancomycin (strain S382, *mreB::mreB‐msfGFP lptD*4213/pDB192) in MM + glu in flow chambers. MreB motion stopped 2 min (vancomycin), 21 min (D‐cycloserine), or 30 min (fosfomycin) after drug addition, as indicated by arrows (see Movies [Link], [Link]).Single‐cell growth of drug‐treated cells as in (A) as a function of time since MreB‐motion stop according to panel (A). Left and middle: Relative increase (left) and single‐cell rates (middle) of volume, mass, surface, and length. Gray asterisks indicate single‐cell mass at lysis. Right: Relative changes in dry‐mass density, surface‐to‐mass ratio, surface‐to‐volume ratio, and width.Single‐cell growth of nondividing *S. enterica* serovar Typhimurium (left) and *V. cholerae* (right) on agarose pads (RDM) containing 1 mM D‐cycloserine. Subset of the same quantities as in (B). Dashed lines indicate nontreated cells.Complex nutrient shift of nondividing cells (S382) growing on agarose pad (MM + 0.02% glucose) in the presence of vancomycin (100 μg/ml). Same quantities as in (B), as a function of time since MreB‐motion stop. Cells were transiently starved for glucose (through addition of 0.5% (w/v) αMG and 0.25% (w/v) 2DG), followed by the addition of excess glucose (1%). Different from (A–C), all compounds were added to the agarose pad as droplets and reached cells by diffusion. Data information: Solid lines + shadings = average ± 2*standard error. Each experiment was repeated twice from a different colony. We analyzed 10–30 single cells for each microscopic experiment. For more details including exact number of cells and absolute values of relative quantities, see Appendix Tables S1 and S2.

We previously showed that *E. coli* expands its cell envelope in proportion to the biomass growth rate on the generation timescale (Oldewurtel *et al*, 2021). To test whether the coordination of envelope growth with biomass growth depends on cell wall insertion, we took time‐lapse movies of *E. coli* cells upon inhibition of cell wall insertion. For absolute quantification of cell shape and cellular dry mass, we immobilized cells in a flow chamber and imaged them through phase‐contrast and quantitative‐phase microscopy as previously described (Oldewurtel *et al*, 2021). To inhibit cell wall insertion, we treated cells with different antibiotics that inhibit peptidoglycan‐precursor synthesis (D‐cycloserine (Lambert & Neuhaus, 1972), fosfomycin (McCoy *et al*, 2003)) or cross‐linking (vancomycin (Barna & Williams, 1984)) (Fig 1). For treatment with vancomycin, which is too large to diffuse through the outer‐membrane pores of wild‐type cells, we used the outer‐membrane‐permeable *lptD*4213 mutant (Sampson *et al*, 1989; Ruiz *et al*, 2005). For accurate image analysis of growing single cells, we prevented cell division by expressing SulA, which inhibits Z‐ring formation (de Boer *et al*, 1990).

First, we grew cells in a minimal medium supplemented with 0.4% glucose (MM + glu) in a flow chamber under constant flow and then switched to a medium containing a cell wall targeting antibiotic at time zero (Fig 1A; see Materials and Methods and Appendix Table S1 for details on this and all other growth experiments). To determine when drug treatment inhibits cell wall insertion, we measured the rotation of the bacterial cytoskeleton MreB‐actin, which depends on cell wall insertion by the multi‐enzyme rod complex (van Teeffelen *et al*, 2011). We therefore conducted our experiments in strains that express a functional fluorescent‐protein fusion to MreB (MreB‐msfGFP) from the native *mreB* locus (Ouzounov *et al*, 2016). MreB rotation was stopped within 2, 21, and 30 min after vancomycin, D‐cycloserine, and fosfomycin treatment, respectively (Fig 1A and Movies [Link], [Link]). For details on this and all other MreB‐motion measurements see also Appendix Table S2. To test whether cell wall insertion was fully arrested, we additionally quantified the incorporation of radioactively labeled cell wall precursor molecules in independent batch experiments (Appendix Fig S1A), by adding ^3^H‐mDAP to a *lysA* mutant, which inhibits the conversion of ^3^H‐mDAP to lysine (Wientjes *et al*, 1985). Cell wall insertion stops 2, 10–20, or 20–40 min after vancomycin, D‐cycloserine, and fosfomycin treatment, in quantitative agreement with our MreB‐based assay.

We then studied surface and mass growth under the microscope. We found that both rates remain almost unperturbed even after the complete cessation of MreB rotation or ^3^H‐mDAP insertion, culminating in sudden cell lysis (Fig 1B and Movies [Link], [Link]). For control experiments without drug, see Appendix Fig S1B and C. Between MreB‐motion arrest and lysis, cells expand their surface area by an additional 15–30% depending on the drug. Previous studies based on hypo‐osmotic shocks demonstrated that the cell envelope cannot possibly stretch elastically by an amount of 15–30% observed here (Buda *et al*, 2016). Therefore, cell wall‐cleaving autolysins must be responsible for extending the area of the cell wall, independent of cell wall insertion.

We also note that both D‐cycloserine and fosfomycin treatment leads to a steady, continuous reduction of MreB activity over the course of about 20 or 30 min prior to arrest, respectively. Yet, surface‐area growth does not change during or after these time frames (Fig 1B).

We observed the same overall behavior when growing cells in a rich defined medium (RDM + glu; Appendix Fig S1D and E): Cell elongation continues at a nearly unperturbed rate by about 20–40% after MreB rotation has stopped, up to sudden cell lysis. The continued growth after MreB‐motion stop can also be observed in the same cell, if taking an MreB time‐lapse movie with a longer time interval (Movie EV7). Finally, consistent with microscopy analysis, drug‐treated batch cultures continue to grow without perturbation by approximately 10–20% (Appendix Fig S1A).

During drug treatment, the rate of cell elongation is slightly reduced in both minimal and rich media (Fig 1B and Appendix Fig S1E). This is likely a necessary response to cell widening, which, in turn, is caused by the lack of rod‐complex activity (Vigouroux *et al*, 2020; Oldewurtel *et al*, 2021). However, the rate of surface growth remains nearly constant and equal to the rate of mass growth.

We also tested the behavior of two other Gram‐negative proteobacteria, *Salmonella enterica* serovar Typhimurium and *Vibrio cholerae*, after treatment with D‐cycloserine (Fig 1C, and Movies EV8 and EV9). Since the *V. cholerae* outer membrane is known to resist lysis (Dörr *et al*, 2015), cell wall damage is identified as sudden bulging. Both bacteria behave similarly to *E. coli*: In *S. enterica*, surface and mass continue to increase at unperturbed rates up to sudden lysis. *V. cholerae* shows a mild reduction of both surface‐ and mass growth rates up to the point of apparent bulging. In conclusion, cell wall expansion continues in the absence of cell wall insertion in different proteobacteria.

Next, we tested whether cells treated with a cell wall insertion inhibitor retain their capacity to modulate the rate of surface growth in synchrony with biomass growth even if the mass growth rate suddenly changes (Oldewurtel *et al*, 2021). We therefore treated cells with vancomycin while also exposing them to a complex nutrient shift, by first starving cells for glucose, through treatment with two glucose analogs (α‐methylglucoside (αMG; Freese *et al*, 1970) and 2‐deoxyglucose (2DG; Wick *et al*, 1957)), followed by the addition of excess glucose (Fig 1D and Movie EV10). For this experiment, we grew cells on an agarose pad and added the different compounds in the form of droplets, which reach cells by diffusion. Since we added vancomycin in excess (100 μg/ml), MreB motion (Movie EV11) stopped well before the reduction of growth rate. Despite a strong, transient reduction in mass growth rate and concomitant variations in cell diameter and mass density, surface area and mass remained nearly perfectly coupled. Mild variations of the surface‐to‐mass ratio immediately after metabolic perturbations are likely due to variations of turgor pressure, as previously demonstrated for multiple nutrient shifts (Oldewurtel *et al*, 2021). From these experiments, we conclude that peptidoglycan synthesis is neither rate‐limiting nor rate‐determining for the global rate of cell wall‐ and cell envelope expansion across different Gram‐negative bacteria.

### Envelope growth responds transiently to variations of endopeptidase levels

**Figure 2:**
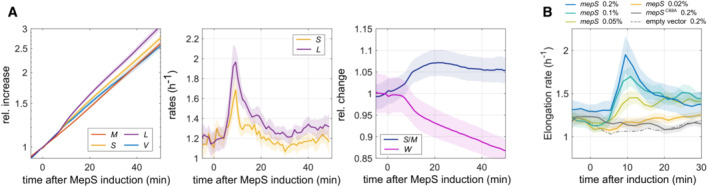
MepS overexpression leads to a transient increase of elongation and surface growth rates Single‐cell growth of nondividing b42 cells (Δ*mepS*/pBAD30‐*mepS*) grown on LB agarose pads as a function of time after the addition of arabinose (0.2%) in the form of a droplet. Left: Relative increase of volume, mass, surface, and length. Middle: Elongation rate and surface growth rate. Right: Relative changes in surface‐to‐mass ratio and width.Elongation rates upon MepS overexpression as in (A) but for different induction levels (0.02–0.2% arabinose) and including empty‐vector control (b55) and inactive mutant (S606 Δ*mepS*/pBAD30‐*mepS* ^C68A^). Data information: Solid lines + shadings = average ± 2*standard error. Each experiment was repeated twice from a different colony. We analyzed about 10–30 single cells for each microscopic experiment. For more details including exact number of cells and absolute values of relative quantities, see Appendix Table S1.

Cell envelope expansion during drug treatment requires the activity of lytic enzymes that cleave peptidoglycan. DD‐endopeptidases are thought to play a central role during cell elongation by cutting peptide bonds between circumferentially oriented glycan strands (Singh *et al*, 2012; Vollmer, 2012; Garde *et al*, 2021). To test the role of DD‐endopeptidases for cell‐surface growth, we strongly overexpressed the DD‐endopeptidase MepS (Singh *et al*, 2012) from a plasmid‐based inducible promoter in the background of a strain deleted for the native *mepS* gene, as confirmed by Western blot (Appendix Fig S2A). We found that rates of cell elongation and surface‐area growth increase rapidly (Fig 2A and Movie EV12), with all single cells showing the same qualitative behavior (Appendix Fig S2B). However, the elongation rate and surface rate increase only transiently (during 5–10 min), while the MepS protein level remains high (Appendix Fig S2A). The effect is dose‐dependent: When inducing *mepS* at reduced levels of arabinose, protein levels are reduced (Appendix Fig S2C) and the burst size decreases (Fig 2B). To investigate whether the elongation burst was caused by endopeptidase activity, we also conducted controls with an empty vector and with an inactive MepS mutant (Singh *et al*, 2012), which both show no elongation burst (Fig 2B). Here, we also confirmed that the inactive mutant was expressed to similar levels as the wild‐type enzyme using Western Blot (Appendix Fig S2C). We will come back to the short duration of the burst further down.

Next, we tested whether the MepS‐caused elongation burst was independent of cell wall insertion. We therefore induced MepS expression 15 min after D‐cycloserine treatment (Appendix Fig S2D). At this point, MreB rotation was stopped in the LB medium (Movie EV13) used in this experiment. Since the arrest of MreB motion is coincident with the arrest of cell wall insertion in minimal medium (Fig 1A and Appendix Fig S1A), cell wall insertion is likely also completely stopped after MreB‐motion stop in LB medium. We thus use MreB‐rotation arrest as a proxy for the time of cell wall insertion arrest in this and subsequent experiments.

Treatment with D‐cycloserine had no detectable effect on the burst size or duration of MepS overexpression (Fig 2A), suggesting that endopeptidase activity is indeed independent of cell wall insertion. However, different from untreated cells, many cells lysed right after the burst (Appendix Fig S2D), in agreement with the requirement of cell wall insertion for the repair of cell wall defects, which likely accumulate upon MepS overexpression (Vigouroux *et al*, 2020).

Finally, we also tested whether cell elongation in the absence of cell wall synthesis required a specific endopeptidase, among the redundantly essential MepM, MepH, and MepS. We thus tested the ability of the corresponding single‐gene deletion mutants to continue cell elongation after D‐cycloserine treatment (Appendix Fig S2E). Single‐gene deletion mutants for these endopeptidases continued to elongate after D‐cyloserine treatment (Appendix Fig S2E), well after MreB motion had arrested, as tested in the *mepS* mutant (around 10 min, Movie EV13), and we did not observe any differences between drug‐treated and untreated cells. Thus, none of the three hydrolases is individually responsible for elongation in the absence of cell wall insertion.

In summary, envelope growth can be transiently accelerated by strong MepS overexpression. However, the observed elongation burst is of short duration. While the rapid inactivation of other autolysins might also contribute to the reduction of envelope growth, the fact that MepS is strongly overexpressed in our experiments, suggests that MepS cannot simply be constitutively active after the elongation burst. Thus, envelope growth is likely not controlled through endopeptidase levels but through the regulation of activity at the single‐enzyme level.

### Local cell wall expansion in response to mechanical forces is independent of cell wall insertion

**Figure 3:**
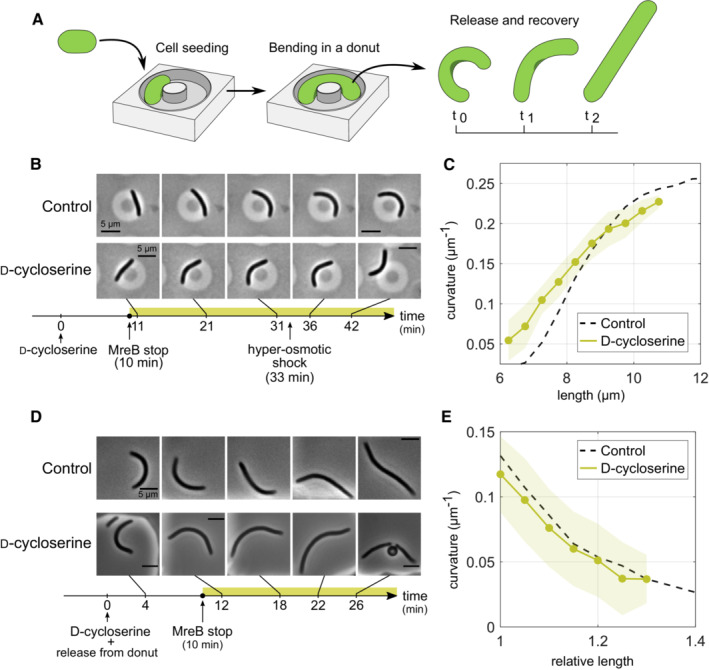
Cells bend and straighten independently of cell wall synthesis AIllustration of bending and straightening of filamentous *E. coli* cells using donut‐shaped microchambers.B, CCell bending: (B) Time‐lapses of bending filamentous cells (S290) in donut microchambers (3% agarose; RDM + glu) and grown without (top) or with (bottom) D‐cycloserine (1 mM) as a function of time since D‐cycloserine treatment, starting > 10 min after D‐cycloserine treatment (the time of MreB‐motion stop, Movie EV14). D‐cycloserine‐treated cells were osmotically shocked (NaCl) after 33 min of treatment, which releases a fraction of cells, demonstrating differential envelope growth during bending (see Appendix Fig S3A for more examples). (C) Change of cell‐centerline curvature as a function of cell length between 10 min after the start of D‐cycloserine treatment and release of cells through osmotic shock (see Appendix Fig S3B for cell length).D, ECell straightening. (D) Time‐lapses of straightening filamentous cells (S290) previously bent in donuts: control (top) and D‐cycloserine treated cells (bottom) as a function of time since simultaneous release from donuts and beginning of drug treatment. (E) Change of centerline curvature as a function of relative length from 10 min after D‐cycloserine treatment (time of MreB‐motion stop). Data information: Solid lines + shadings = average ± 2*standard deviation. Each experiment was repeated at least twice from a different colony. We analyzed about 10–70 single cells for each experiment. For more details including exact number of cells and absolute values of relative quantities and replicates, see Appendix Table S1.

Rod‐shaped bacteria grow plastically into curved shapes when physically bent (Takeuchi *et al*, 2005; Amir *et al*, 2014; Wong *et al*, 2017), for example when confined into donut‐like microchambers (Fig 3A). Once released, bent cells straighten plastically during growth (Takeuchi *et al*, 2005; Amir *et al*, 2014). Both phenomena can be reconciled through a model, in which the local rate of cell envelope growth differentially increases with areal mechanical strain, that is, the degree of mechanical cell envelope stretching (Wong *et al*, 2017): During bending, mechanical strain is increased on the outer side of the bent cell as compared to the inner side (Wong *et al*, 2017), which can thus explain growth into a curved cell shape. During straightening, mechanical strain is increased on the inner side of the curved shape (Wong *et al*, 2017), which can explain cell straightening. Here, we investigated whether cells retain the ability of strain‐dependent differential growth during bending or straightening in the absence of cell wall insertion (Fig 3).

First, we confined filamentous cells grown in a rich defined medium (RDM + glu) into donut‐shaped microchambers about 20 min after inducing filamentation and about 10 min after starting treatment with D‐cycloserine (1 mM). Cell wall insertion is likely arrested at that time point since MreB rotation stops 10 min after D‐cycloserine treatment in RDM + glu (Movie EV14). Yet, cells elongate and curve just like untreated cells, up to the point of cell lysis, as observed in time‐lapses (Fig 3B, Appendix Fig S3A and Movie EV15) and in both average centerline curvature (Fig 3C) and cell elongation (Appendix Fig S3B).

Differential growth of untreated cells can be demonstrated by physically removing cells from donuts (Takeuchi *et al*, 2005; Wong *et al*, 2017). To extract D‐cycloserine‐treated cells quickly from donuts, prior to lysis, we applied a hyperosmotic shock (0.5 M NaCl) after approximately 30 min of drug treatment by adding a droplet of concentrated NaCl solution to the top of the agarose pad, which reaches the cells by diffusion within minutes. Due to a combination of osmotic cell shrinkage and added liquid, a fraction of cells left the donut chambers, while retaining a bent shape (Fig 3B and Appendix Fig S3A). Thus, these cells had experienced differential and plastic envelope growth just like the control cells. Since MreB motion was stopped at the time of loading cells into donuts, cells responded to differential mechanical stress or strain through differential envelope growth in the absence of cell wall insertion.

Next, we investigated whether cell wall insertion is also dispensable for straightening from bent conformations. To that end, we first grew filamentous cells in donut‐shaped microchambers without drug treatment, then released cells from confinement, and followed their shape evolution in deep agarose‐based growth chambers containing 1 mM D‐cycloserine under the microscope (Fig 3A). Cells continue to straighten well after 10 min (Fig 3D and Movie EV16), the time of MreB‐motion stop in RDM + glu (Movie EV14). While cell elongation is slightly reduced (Appendix Fig S3C), cell‐centerline curvature decreases with cell length just like in untreated control cells (Fig 3E). Here, we started our quantitative analysis 10 min after the beginning of drug treatment to study straightening after the arrest of cell wall insertion. These experiments demonstrate that cell wall insertion has no apparent influence on cell bending and straightening, contrary to previous suggestions by us and others (Ursell *et al*, 2014; Wong *et al*, 2017).

### Cell widening in response to turgor‐pressure changes is independent of cell wall insertion

**Figure 4:**
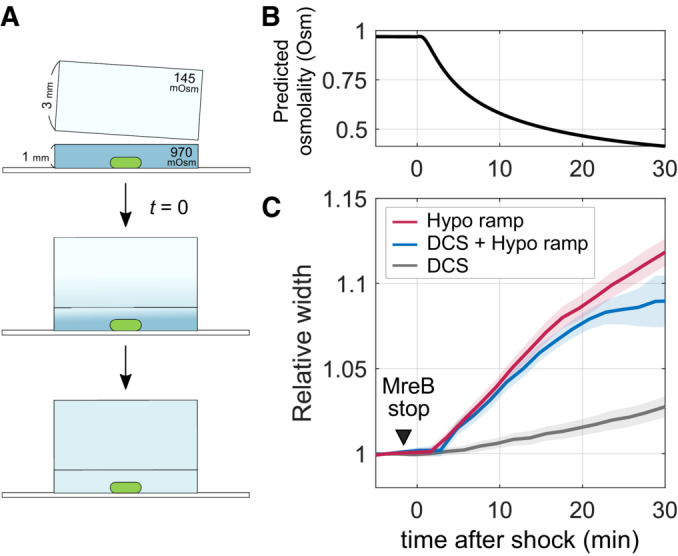
Cell width increases upon hypo‐osmotic ramp in the absence of cell wall synthesis Cartoon of a hypo‐osmotic ramp experiment. Cells are initially grown on a high‐osmolality agarose pad and a second pad of low osmolality is added. This leads to a diffusion‐limited decrease of osmolality from 970 to 351 mOsm, with a decrease of about 500 mOsm occurring during the first 20 min (see B).Predicted osmolality at the position of the cells based on one‐dimensional diffusion model for the hypo‐osmotic ramp illustrated in (A).Relative change of cell width of nondividing cells (S257) grown in LB during combined D‐cycloserine treatment (5 mM) and a hypo‐osmotic ramp as in A (DCS + Hypo ramp), as compared to cells exposed to the hypo‐osmotic ramp only (Hypo ramp) or treated with D‐cycloserine only (DCS). D‐cycloserine was contained in both agarose pads, and treatment started 15 min prior to the shock. The time of MreB‐rotation stop is indicated by an arrow (See Movie EV17). Data information: Solid lines + shadings = average ± 2*standard error. Each experiment was repeated at least twice from a different colony. We analyzed about 40 single cells for each experiment. For more details including exact number of cells and absolute values of relative quantities, see Appendix Table S1.

We previously demonstrated that *E. coli* cells change their cell width plastically and in a dose‐dependent manner in response to changes in cytoplasmic osmotic pressure (Oldewurtel *et al*, 2021). To that end, we conducted diffusion‐limited NaCl‐based hypo‐osmotic ramps similar to the one illustrated in Fig 4A. Here, an agarose pad of low salt concentration is added on top of an agarose pad of high concentration, which leads to the continuous decreases of medium osmolality at the position of the coverslip by about 500 mOsm over the course of 20 min (Fig 4B). Since medium osmolality keeps on changing during this time frame, cytoplasmic turgor is likely elevated throughout the ramp (Oldewurtel *et al*, 2021).

During the 500 mOsm ramp of Fig 4, control cells (labeled as “Hypo ramp” in Fig 4C) widen by about 8%, in agreement with previous observations (Oldewurtel *et al*, 2021). The 8% increase in width cannot be explained by elastic stretching alone but requires plastic cell widening, since cells exposed to a sudden hypo‐osmotic shock of similar magnitude (520 mOsm) show instantaneous elastic widening of only about 2.5% (Oldewurtel *et al*, 2021).

Here, we wondered whether plastic cell widening occurred independently of cell wall insertion. In analogy to our experiments on cell bending, we thus grew cells on agarose pads containing D‐cycloserine. Since it is known that high salt reduces the susceptibility to antibiotics through increasing efflux pump expression (Zhu & Dai, 2018), we used a higher D‐cycloserine concentration (5 mM), which arrests MreB rotation within 15 min after drug treatment (Movie EV17) similar to cells grown at low salt concentration. We then exposed these cells to the same hypo‐osmotic ramp as above but only 15 min after the beginning of D‐cycloserine exposure (labeled as “DCS + Hypo ramp” in Fig 4C, see also Movie EV18 and Appendix Fig S4). During the first 20 min after starting the hypo‐osmotic ramp, cells increase their width by the same amount as untreated cells (8%) and much faster than D‐cycloserine‐treated cells that were not exposed to the hypo‐osmotic ramp (curve “DCS” in Fig 4C). Since cells are continuously treated with D‐cycloserine from 15 min prior to the ramp, cell wall synthesis is likely arrested during the time of cell widening. Thus, cells plastically increase their cell width independently of cell wall insertion, and in direct response to elevated osmotic pressure.

## Discussion

In conclusion, we found that the global rate of cell envelope growth and the differential rates of bending, straightening, and widening are controlled independently of cell wall insertion. Instead, the global rate of envelope expansion is robustly coupled to biomass growth, even if the mass growth rate changes through nutrient shifts. Furthermore, differential rates of envelope expansion likely respond to areal mechanical strain (Wong *et al*, 2017). These findings contrast with widely held previous hypotheses that cell wall insertion is rate‐limiting for both global and local cell envelope growth. The independence of the global rate of envelope growth on cell wall insertion is roughly conserved across distant Gram‐negative proteobacteria (Fig 1B and C). However, the behavior is qualitatively different in the Gram‐positive *Bacillus subtilis*, where inhibition of cell wall insertion leads to a strong reduction and decoupling of surface‐ and mass growth (Kitahara *et al*, 2022).

While cell wall insertion is not rate‐limiting for both global and local rates of envelope growth, it is required for the maintenance of cell wall integrity, as demonstrated by rapid cell lysis after drug treatments (Fig 1).

### Cell wall insertion does likely not affect the growth of any envelope component

The observation that cell wall synthesis inhibition does not affect surface‐area growth implies that the outer membrane continues to grow in surface area at an unperturbed rate independently of cell wall insertion. Whether its structural or mechanical properties are affected, remains to be studied in the future. Additionally, we reason that the inner membrane must also continue to grow because cells do not show any sign of plasmolysis. The latter should be visible if the cytoplasmic volume did not increase while the total volume increases by 30% (Fig 1B). A mild reduction in cytoplasmic membrane growth would be harder to detect and can thus not be fully ruled out, but given the fact that cells continue to grow at unperturbed rates in surface area and mass after drug treatment suggests to us that cell physiology, and thus also cell envelope geometry and membrane integrity, are likely not severely perturbed.

### The role of cell wall insertion for cell shape

If cell wall insertion is not required for cell envelope expansion, what then is the role of cell wall insertion for cell shape? Cell wall insertion is responsible for the maintenance of cell width and for the circumferential orientation of the peptidoglycan mesh on long timescales, notably through the MreB‐based rod complex (Turner *et al*, 2013). Thus, cell wall insertion affects the spatial pattern and orientation of autolytic activity through their common substrate, even in the absence of direct interactions between rod complex and autolysins. Direct interactions between the rod complex and autolysins have indeed not been reported in Gram‐negative bacteria (Rohs & Bernhardt, 2021). We therefore speculate that indirect interactions through the cell wall substrate might also be responsible for short‐term spatial correlations between cell wall expansion and MreB‐actin (Ursell *et al*, 2014). Independently of the type of interaction, the process of cell wall insertion is not rate‐limiting for the overall rate of envelope growth (Fig 1), nor does it dominate the spatial distribution of autolytic activity if compared with the role of mechanical forces (Figs 3 and 4 and ref. Wong *et al*, 2017).

Different from the rod complex, class‐A penicillin‐binding proteins (aPBPs) are not required for cell shape but are important for cell wall integrity (Vigouroux *et al*, 2020). Here, the causality is likely inverted: Cell wall‐cleaving autolysins create local pores in the cell wall, which are then filled through aPBP activity (Lai *et al*, 2017; Vigouroux *et al*, 2020). aPBP‐based cell wall insertion might then also explain part of the correlations between differential cell wall insertion and differential envelope growth previously observed in the intrinsically curved or helical bacteria *Vibrio cholerae* (Bartlett *et al*, 2017), *Helicobacter pylori* (Taylor *et al*, 2020), and *Caulobacter crescentus* (Cabeen *et al*, 2009). Accordingly, differential cell wall insertion would not be a cause but the consequence of autolytic cell wall expansion. Consistently, *V. cholerae* cells bend in the absence of rod‐complex or aPBP activities (Martin *et al*, 2021).

### How does autolytic activity relate to biomass growth?

How does the global rate of cell wall expansion respond to biomass growth, if not through cell wall synthesis and insertion as previously proposed (Harris & Theriot, 2016)? One attractive possibility: The abundance of cell wall‐cleaving autolysins might have an important regulatory role, notably for the DD‐endopeptidase MepS in *E. coli*. This enzyme undergoes constant proteolytic degradation (lifetime of less than 3 min; Singh *et al*, 2012, 2015), which allows rapid modulation of enzyme levels. Indeed, we observed a rapid increase in cell elongation and surface growth upon overexpression of MepS (Fig 2). However, the effect is relatively short (5–10 min), while protein levels are continuously strongly overexpressed. This experiment suggests that MepS enzymes are regulated at the enzyme level, through an unknown signal or regulatory protein. Other autolysins such as lytic transglycosylases might also contribute to cell elongation.

### How does autolytic activity respond to cell geometry and therefore to differential mechanical strain?

We had previously speculated that differential envelope growth might be caused by strain‐sensing pairs of aPBPs and their cognate outer‐membrane‐based activators (LpoA or LpoB; Wong *et al*, 2017). Those pairs span the periplasm and might insert peptidoglycan in response to cell wall pore size (Paradis‐Bleau *et al*, 2010; Typas *et al*, 2012; Vigouroux *et al*, 2020). Here, we found that cell wall synthesis, including the activity of aPBPs, is not responsible for differential cell envelope growth. This would still allow for the possibility that aPBPs and their cognate outer‐membrane protein recruit cell wall‐cleaving enzymes to sites of increased strain. However, DD‐endopeptidases could also act as strain sensors or stress sensors by themselves. Notably, the *V. cholerae* DD‐endopeptidase ShyA is found in open (possibly active) or closed (inactive) conformations (Shin *et al*, 2020). Transitions between the two conformations could be induced by changes in cell wall architecture, pore size, and thus by strain or stress (Shin *et al*, 2020). However, further work is needed to determine the causal relationship between autolytic activity and envelope mechanics.

## Materials and Methods

### Plasmid and strain construction

Plasmids, strains, and primers are indicated in Appendix Tables S3–S5, respectively.

#### Plasmid construction

pBC04 (P_BAD_‐*mepS*): *mepS* was cloned under an arabinose‐inducible promoter in the pBAD30 vector. *mepS* was amplified from *E. coli* genomic DNA using the primers P001 containing a ribosome‐binding site and a SacI restriction site and P002 containing a XbaI restriction site. After digestion, the *mepS*‐containing SacI‐XbaI fragment was ligated with the pBAD30 fragment cleaved by SacI and XbaI.

pKY01 (P_BAD_‐*mepS* ^C68A^): Two fragments based on pBC04 were amplified using the following sets of primers P003, P004 and P005, P006, respectively. The two vector fragments were combined by Gibson assembly.

#### Strain construction

All strains except for EB06 were generated by straight‐forward P1 phage transduction and/or plasmid transformation as indicated in Appendix Table S4. A kanamycin resistance cassette was removed using pCP20 by classical FLP recombination protocol (Baba *et al*, 2006) as indicated.

EB06 (MG1655 *lptD*4213) was constructed as follows: We transduced MG1655 with P1 phage prepared from NR693 (MC4100 *lptD* 4213, *carB*:Tn10; Ruiz *et al*, 2005) and selected for growth on carbenicillin and found mutants that did not grow on MacConkey agar. One of the mutants, EB03 (MG1655 *lptD* 4213, *carB*:Tn10), was transduced a second time with the same P1 phage and selected for growth on minimal media supplemented with glucose, which requires *carB*, to obtain EB06.

### Growth conditions and microscopy

Cell cultures were grown from an individual colony in LB Miller medium (LB) to the exponential phase with an appropriate selection of antibiotics. Strains with plasmids were grown with 50 μg/ml carbenicillin during overnight growth. For microscopy, cells were then washed and diluted into LB, MOPS Rich Defined Medium (RDM; MOPS EZ Rich Defined Medium, M2105, Teknova), or defined MOPS minimal medium (MM) (MOPS Minimal Media Kit, M2106, Teknova) without antibiotic. We added 0.02–0.4% (w/v) glucose to RDM and MM, depending on the experiment (Appendix Tables S1 and S2). Cells were grown at 30°C in liquid media in a shaking incubator. To monitor single‐cell growth without division, we inhibited cell division either by inducing *sulA* with 1 mM IPTG from plasmid pDB192 or by adding 10 μg/ml aztreonam (Sigma‐Aldrich, A6848) shortly before microscopy (Appendix Tables S1 and S2). To inhibit cell wall synthesis, we treated cells with vancomycin (Fisher Scientific, BP2958‐1), Fosfomycin (Sigma‐Aldrich, P5396‐1G), or D‐cycloserine (Sigma‐Aldrich, 30020‐1G). To perform microscopy, we used three different supports, flow chambers, agarose pads, or donut‐shaped chambers, as described in the following. During sample preparation and subsequent imaging, the temperature was maintained at 30°C.

#### Flow chamber

For the microscopy measurements of cell growth during antibiotic treatment and their controls presented in Fig 1A and B, and Appendix Fig S1B and C, we used sticky‐slide I Luer 0.2 (ibidi) channels, on top of 24 × 60 mm coverslips (Corning No 1.5), which were precoated with APTES ((3‐Aminopropyl)triethoxysilane, Sigma‐Aldrich, A3648‐100ML), as previously described in detail (Oldewurtel *et al*, 2021). After loading cells, we provided MM + glu medium at a constant flow rate of 50 μl/min via connected silicone tubings using a syringe pump (70‐4501, Harvard Apparatus). To conduct instantaneous drug treatments, we prepared two syringes (MM + glu with and without drug) connected to a Y‐junction about 5 cm upstream of the channel, which allows to switch the flow from the medium without drug to the one with drug within seconds.

#### Agarose pad

For the microscopy experiments shown in Fig 1C and D, Appendix Fig S1D and E, Fig 2, Appendix Fig S2, and Fig 4, we immobilized cells on agarose pads prepared from the fresh culture medium and 1% UltraPure Agarose (16500–500, Invitrogen), by sandwiching cells between agarose pad and the coverslip of circular dishes (ibidi). To expose cells to different compounds, we either contained the compound in pads or added concentrated compound solution on top of the pads in the form of a droplet to achieve the final concentrations indicated in Appendix Tables S1 and S2. Agarose pads have a height of 1.5 mm (75 or 150 μl volume). Thus, compounds added in the form of droplets reach cells within minutes according to a one‐dimensional diffusion model. For example, 1% of the final vancomycin concentration is reached within approximately 5 min, based on an estimated diffusion coefficient of 2.83·10^−6^ cm^2^/s (Stewart, 1996).

For the complex nutrient shift experiment shown in Fig 1D, we first grew S382 cells in MM + 0.02% glucose. At the time of the first arrow in Fig 1D, we added 100 μg/ml vancomycin and 0.5% (w/v) alpha‐methylglucose (αMG; Sigma‐Aldrich, M9376) and 0.25% (w/v) 2‐deoxyglucose (2DG) (Sigma‐Aldrich, D8375‐1G) to the pad as drop and then at the time of the second arrow we added 1% glucose to the pad.

For the MepS induction experiments in Fig 2, cells were grown in LB, and 0.02–0.2% arabinose was added to the pad as a droplet. For the experiment in Fig 2A, prior to sample preparation, cells were grown in LB liquid medium supplemented with 0.2% glucose to repress MepS expression. Cells were washed by centrifugation and resuspension in LB directly before placing cells under the LB agarose pad.

For hypo‐osmotic ramp experiments (Fig 4), S257 cells were first grown on an LB agarose pad (osmolality: 970 mOsm; dimension: 7 × 7 mm; height: 1 mm). To perform a diffusion‐based hypo‐osmotic ramp, another LB agarose pad (osmolality: 145 mOsm; dimension: 7 × 7 mm; height: 3 mm) was put on top of the higher‐osmolality agarose pad as described (Fig 4A). This leads to a slow decrease in NaCl concentration. Osmolality of the liquid medium for pad preparation was measured by a freezing point osmometer (i Osmometer basic, Loeser) and adjusted using NaCl.

#### Donut‐shaped microchamber

For bending and straightening experiments (Fig 3) we used donut‐shaped microchambers (outer diameter = 8 μm, inner diameter = 2 μm) as previously described (Wong *et al*, 2017).

For bending experiments (Fig 3B and C), S290 cells were preincubated in RDM + glu (to an OD ~ 0.2–0.3). Eighteen minutes prior to loading cells to the donuts, we added 1 mM IPTG to induce SulA. Ten minutes later, we added 1 mM D‐cycloserine to inhibit cell wall synthesis. Cells were subsequently transferred onto a 3% agarose pad (RDM + glu, 1 mM D‐cycloserine, 1 mM IPTG) containing donut‐shaped microchambers. Twenty‐five minutes after the start of microscopy a droplet of 5 M NaCl solution was added on top of the agarose microchambers (final concentration = 0.5 M) to condense cells and possibly free them from the donut walls. The hyperosmotic shock thus allowed us to demonstrate that cells are intrinsically bent after growth in the donut, rather than being elastically bent.

For straightening experiments (Fig 3D and E), S290 cells were grown in donuts as above but without D‐cycloserine. After incubation for 45–60 min in the donuts, cells were released from the donuts and transferred into agarose‐based growth chambers (RDM + glu, 1 mM D‐cycloserine, 1 mM IPTG + 1 mM D‐cycloserine; dimension: 30 × 30 μm and 25 μm deep) as described (Wong *et al*, 2017). Only cells positioned in the focal plane of the microscope were considered for analysis.

### Microscopy and image analysis

Microscopy experiments were performed on two different setups: All microscopy except for bending and straightening experiments was carried out using a Nikon Ti‐E inverted phase‐contrast, epi‐fluorescence microscope and an additional module for spatial light interference microscopy (SLIM; Wang *et al*, 2011) as described (Oldewurtel *et al*, 2021). The microscope is equipped with a temperature chamber (Stage Top Incubator, Okolab) set at 30°C, a Nikon Plan Apo 100× NA 1.45 Ph3 objective, a solid‐state light source (Spectra X, Lumencor Inc., Beaverton, OR), a multiband dichroic (69002bs, Chroma Technology Corp., Bellows Falls, VT), and excitation (485/25) and emission (535/50) filters for GFP imaging. Epi‐fluorescence images were acquired with an sCMOS camera (Orca Flash 4.0, Hamamatsu), while phase‐contrast and quantitative‐phase images were obtained with another CMOS camera (DCC3260M, Thorlabs). We took phase‐contrast and quantitative‐phase images for simultaneous measurements of the shape and dry mass of single cells. The two‐dimensional contours of single cells were obtained from phase‐contrast images using the Morphometrics package (Ursell *et al*, 2017), after prior calibration based on the membrane stain FM4‐64 (Oldewurtel *et al*, 2021). Mass measurements were conducted by spatial light interference microscopy (SLIM) and simulation‐based image analysis as previously described in detail (Oldewurtel *et al*, 2021). In brief, for every phase image, we obtained 6 phase‐contrast images, each taken with a different phase delay between scattered and unscattered light (differing by π/2). Those then allow the calculation of the phase image through a simple algebraic relationship. From those images, the integrated phase is obtained by summing up all positive pixel values. The integrated phase is corrected for optical artifacts of the microscope by comparing the integrated phase signal with the corresponding signal from computational image simulations. The latter are obtained for every real image, based on cell contour and on the parameters of the microscope. For details see the supplementary material of Oldewurtel *et al* (2021).

For the calculation of growth rate from time‐lapse microscopy, we filtered time traces of mass, length, width, surface, and volume using a Gauss filter of standard deviation σ as indicated in Appendix Table S1. Relative rates $\lambda_{X}=$ $d\left(\log X\right)/dt$ with X=S,W,L,M were calculated as

$$\lambda_{X}\left(t_{i+1/2}\right)=\frac{2\left(X_{i+1}-X_{i}\right)}{\left(X_{i+1}+X_{i}\right)\Delta t_{i}},$$

where $t_{i+1/2}=0.5\left(t_{i}+t_{i+1}\right).$ Before display, rates were smoothened with the same filter used for quantities *X*.

For quantitative MreB analysis, we took epi‐fluorescence images of MreB‐msfGFP close to the bottom of the cells (about 250 nm below the central plane of cells) every 1 or 2 s depending on the experiments (See Appendix Table S2 for details including time intervals and movie durations). We then detected and tracked MreB filaments using the Fiji plugin TrackMate (Tinevez *et al*, 2017) using the Laplacian of Gaussians (LoG) detector with a 0.15 μm spot diameter and a linear motion LAP tracker (search radius 0.06 or 0.12 μm and number of allowed gaps 1 or 2 for 1 s‐interval movies or 2 s‐interval movies, respectively). *x*‐ and *y*‐coordinates of tracks were smoothened by a Savitzky–Golay filter with a span of seven steps. To restrict the analysis to persistent tracks, we considered all tracks of at least seven steps and a minimum end‐to‐end distance of 0.2 μm. Additionally, we took a phase‐contrast image to measure 2D cell area using the Morphometrics package (Ursell *et al*, 2017) for cell segmentation. To measure the density of detected MreB filaments times average MreB speed, which represents the total activity of MreB‐based cell wall insertion (termed “MreB activity”), we calculated the sum of all MreB track lengths during the observation time and divided by total segmented cell area and time. Based on visual inspection of movies, persistent MreB rotation is stopped once the MreB activity underpasses a threshold value of 0.08 μm^−1^ min^−1^ (see Fig 1A). This is the time used for the determination of MreB‐motion stop in all MreB‐msfGFP‐based experiments (shown explicitly in Fig 1A and Appendix Fig S1D).

Microscopy for bending and straightening experiments (Fig 3) was performed as described (Wong *et al*, 2017) using a Zeiss (Jena, Germany) Axiovert 200 inverted microscope. The microscope is equipped with an enclosing custom‐made incubation chamber (set to 30°C), an AxioCam 503 mono charge‐coupled‐device (CCD) camera (Zeiss, Jena, Germany) and a Zeiss EC Plan‐Neofluar 40×/0.75‐NA objective. Cell elongation and curvature were analyzed using the MicrobeJ plugin (Ducret *et al*, 2016) of ImageJ.

### Radioactive ^3^H‐mDAP labeling

S458 and S381 cells were grown in MM + glu supplemented with 0.4 mM lysine following the method described in Wientjes *et al* (1985). OD600 was kept below 0.1 for more than 10 doublings at 30°C. We split the culture into “hot culture” for radioactivity measurement and “cold culture” for OD measurement. Then, we added 1 mM IPTG to both cultures and 5 μCi/ml of ^3^H‐mDAP (MT‐1556, Moravek) to hot culture at *t* = −30 min in Appendix Fig S1A. Afterwards, each culture was split into “control culture” and “drug culture”. We added 1 mM D‐cycloserine or 500 μg/ml Fosfomycin to the S458 drug cultures and 100 μg/ml vancomycin to the S381 drug culture at *t* = 0 min. For radioactivity measurements, samples of 200 μl of each hot culture were taken and boiled in 4% SDS for at least 1 h. After cooling the SDS samples to room temperature, they were filtered through 0.22 μm membrane filters (Millipore GSWP02500). The filters were then washed with 20 ml distilled water three times, collected into scintillation count vials and treated with 400 μl of 10 mg/ml lysozyme at 37°C for at least 2 h, and finally dissolved in 5 ml scintillation cocktail (Filter‐CountTM, Perkin Elmers) overnight. Eventually, counts per minute due to radioactive decay of ^3^H were measured in a scintillation counter (TriCarb, Perkin Elmer). For OD measurements, we took a sample of 0.5 ml from cold cultures and measured OD600.

### Western blot for MepS

100 ml cell culture was grown in LB at 30°C to an OD600 ≈ 0.3. Cells were collected by centrifugation (7,000 × *g* for 5 min at 4°C), and pellets were resuspended in 10 ml of lysis buffer (50 mM Tris–HCl pH 7.5, 100 mM NaCl and a protease inhibitor cocktail (Thermo Scientific)) and flash‐frozen in liquid nitrogen. After a soft thawing on ice, cells were disrupted by sonication (Misonix S‐4000; alternating 3 cycles of 30‐s ON with 40% amplitude and 15‐s OFF to cool down the sample). The lysates were centrifuged (7,000 × *g* for 5 min at 4°C) to remove unbroken cells. The supernatant was collected and centrifuged for 1 h at 100,000 × *g* at 4°C. The pellet (membrane fraction) was suspended in 100 μl of lysis buffer. Protein concentration was determined using a Bradford‐based colorimetric assay (Bio‐Rad 5000006). Prior to SDS–PAGE loading, the samples were diluted in 4× Laemmli sample buffer and concentrations were adjusted to load 6 μg of membrane proteins per lane. The samples were separated by SDS–PAGE with a 4–20% polyacrylamide (Mini PROTEAN TGX gel, Bio‐Rad) and transferred onto nitrocellulose membranes (Bio‐Rad). Membranes were blocked by 3% milk in 1× TBS‐T (Tris, NaCl, Tween‐20) for 1 h at room temperature and then incubated overnight at 4°C with MepS primary antibody (1:1,000 in 1× TBS‐T Milk 1%). The affinity‐purified MepS primary antibody (polyclonal) was generated by ProteoGenix based on the peptide CMGKSVSRSNLRTGD (corresponding to the amino acids 120–133 of MepS) in rabbits. Membranes were washed three times with 1× TBS‐T for 5 min and incubated for 1 h at room temperature with the secondary antibody (Goat Anti‐Rabbit, Bio‐Rad) coupled with horseradish peroxidase (HRP; 1:3,000 in 1× TBS‐T). Prior to signal detection, membranes were washed three times with 1× TBS‐T for 5 min and covered with ECL reagent (Thermofisher, 34580).

## Author contributions

**Enno R Oldewurtel:** Conceptualization; data curation; formal analysis; investigation; visualization; writing – original draft; project administration; writing – review and editing. **Yuki Kitahara:** Conceptualization; data curation; formal analysis; investigation; visualization; writing – original draft; project administration; writing – review and editing. **Baptiste Cordier:** Data curation; formal analysis; investigation. **Richard Wheeler:** Data curation; formal analysis; investigation. **Gizem Özbaykal:** Data curation; formal analysis; investigation. **Elisa Brambilla:** Investigation. **Ivo Gomperts Boneca:** Funding acquisition. **Lars D Renner:** Conceptualization; data curation; formal analysis; funding acquisition; investigation; visualization; methodology; writing – original draft; writing – review and editing. **Sven van Teeffelen:** Conceptualization; supervision; funding acquisition; writing – original draft; project administration; writing – review and editing.

## Disclosure and competing interests statement

The authors declare that they have no conflict of interest.

## Supporting information



Appendix

Movie EV1

Movie EV2

Movie EV3

Movie EV4

Movie EV5

Movie EV6

Movie EV7

Movie EV8

Movie EV9

Movie EV10

Movie EV11

Movie EV12

Movie EV13

Movie EV14

Movie EV15

Movie EV16

Movie EV17

Movie EV18

## Data Availability

Relevant information for all experiments and for plots of relative quantities are found in Appendix Tables S1 and S2. Single‐cell data are available at the Zenodo data repository (DOI:10.5281/zenodo.7783477).
