# Supplementary material for: Cell envelope growth of Gram‐negative bacteria proceeds independently of cell wall synthesis
Source: EMBO J. 2023 Jun 1;42(14):e112168. doi: 10.15252/embj.2022112168 (PMC10350831; doi:10.15252/embj.2022112168)
Supplement: Supplementary file 1 — Appendix [file EMBJ-42-e112168-s005.pdf]

Appendix

Cell envelope growth of Gram-negative bacteria proceeds independently of cell wall synthesis

Enno R. Oldewurtel[1†], Yuki Kitahara[1,2,3†*], Baptiste Cordier[1], Richard Wheeler[4], Gizem Özbaykal[1,2,5], Elisa Brambilla[1], Ivo Gomperts Boneca[4], Lars D. Renner[6*], and Sven van Teeffelen[1,3*]

Table of Contents

Supplementary Tables 2

Appendix Table S1: Detailed information for single-cell growth experiments by SLIM. 2

Appendix Table S2: Detailed information of MreB experiment by fluorescence microscopy 3

Appendix Table S3: Plasmid list 4

Appendix Table S4: Strain list 4

Appendix Table S5: Primer list 4

Supplementary References 5

Supplementary Figures 6

Appendix Table S1: Detailed information for single-cell growth experiments by SLIM.

This table includes experimental types, corresponding figures, used strains, growth media, absolute values of relative quantities, cell numbers, and replicates. See description of columns below the table.

Experiment	Figures	Strain	Medium #1	Medium #2	Δt (min)	σ (steps)	t_{exp} (min)	t_{drug} (min)	t_{MreB} (min)	ρ (g/mL)	S/M (μm²/pg)	S/V (1/μm)	W (μm)	n_{cells}	support	biological replicates
D-cycloserine treatment in minimal medium	1A, 1B	S257	MM+glu	+ D-cycloserine 1 mM	2.2	1	-41 (s)	-21	0	0.322	17	5.47	0.81	31	flow chamber	2 + Fig. S1E for RDM+glu
Fosfomycin treatment in minimal medium	1B	S257	MM+glu	+ Fosfomycin 500 μg/mL	2.2	1	-51 (s)	-30	0	0.331	16.7	5.51	0.8	23	flow chamber	2 + Fig. S1E for RDM+glu
Vancomycin treatment in minimal medium	1B	S382	MM+glu	+ Vancomycin 100 μg/mL	2	1	-23 (s)	-2	0	0.337	16.1	5.44	0.82	14	flow chamber	2 + Fig. S1E for RDM+glu
control in minimal medium	S1B	S257	MM+glu	NA	2	1	-10 (s)	NA	NA	0.332	16.9	5.63	0.8	35	flow chamber	2
control in minimal medium	S1C	S382	MM+glu	NA	2	1	-10 (s)	NA	NA	0.335	16.2	5.44	0.85	21	flow chamber	2
D-cycloserine treatment in rich medium	S1E	S257	RDM+glu	+ D-cycloserine 1 mM	1.1	1	-24 (s)	-13†	0	0.255	15.8	4.02	1.06	60	pad	§
Fosfomycin treatment in rich medium	S1E	S257	RDM+glu	+ Fosfomycin 500 μg/mL	1.1	1	-22 (s)	-15†	0	0.259	15.4	4	1.07	81	pad	§
Vancomycin treatment in rich medium	S1E	S382	RDM+glu	+ Vancomycin 100 μg/mL	1.3	1	-11 (s)	-5†	0	0.252	15.2	3.83	1.15	18	pad	§
Single cell growth without drug (*S. enterica*	1C	*S. enterica* serovar Typhimurium SL1344	RDM	NA	1	0.5	0	NA	NA	0.317	17.5	5.54	0.8	21	pad	2
D-cyclcoserine treatment to *S. enterica*	1C	*S. enterica* serovar Typhimurium SL1344	RDM + 1 mM D-cycloserine	NA	1	0.5	0	0	NA	0.301	17.9	5.37	0.81	32	pad	2
Single cell growth without drug (*V. cholerae*	1C	*V. cholerae* E7946	RDM	NA	1	0.5	0	NA	NA	0.287	21.9	6.25	0.71	23	pad	2
D-cyclcoserine treatment (*V. cholerae*)	1C	*V. cholerae* E7946	RDM + 1 mM D-cycloserine	NA	1	0.5	0	0	NA	0.279	22.1	6.15	0.71	33	pad	2
complex nutrient shift	1D	S382	MM+glucose (0.02%)	#2: + vancomycin 100 μg/mL, 0.5% αMG, 0.25% 2DG	1	1	-58 (s)	-9, 36††	0	0.318	17	5.4	0.84	77	pad	2
MepS overexpression	2A, S2B	b42	LB¶	+ 0.2% arabinose	1	0.5	-10 (a)	NA	NA	0.247	13.9	3.42	1.28	13	pad	2
MepS induction	2B	b42	LB	+ 0.2% arabinose	2	0.5	-13 (a)	NA	NA	0.248	14.4	3.56	1.21	12	pad	2
MepS induction	2B	b42	LB	+ 0.1% arabinose	2	0.5	-24 (a)	NA	NA	0.25	14.3	3.58	1.16	14	pad	2
MepS induction	2B	b42	LB	+ 0.05% arabinose	2	0.5	-24 (a)	NA	NA	0.221	16	3.53	1.19	31	pad	2
MepS induction	2B	b42	LB	+ 0.02% arabinose	2	0.5	-24 (a)	NA	NA	0.206	15.4	3.16	1.34	25	pad	2
MepS induction	2B	S606	LB	+ 0.2% arabinose	2	0.5	-15 (a)	NA	NA	0.23	15.8	3.62	1.19	26	pad	2
MepS induction	2B	b55	LB	+ 0.2% arabinose	2	0.5	-25 (a)	NA	NA	0.219	16.1	3.51	1.21	28	pad	2
MepS induction during D-cycloserine	S2D	b183	LB+ 1 mM D-cycloserine	+ 0.2% arabinose	2	0.5	0 (a)	0	11	0.236	14.4	3.4	1.28	20	pad	2
D-cycloserine treatment (*ΔmepS*)	S2E	EL54	LB+ 1 mM D-cycloserine	NA	2	NA	0 (a)	0	NA	0.213	17	3.61	1.2	35	pad	2
Single cell growth without drug (*ΔmepS*)	S2E	EL54	LB	NA	2	NA	0 (a)	NA	NA	0.251	15	3.75	1.17	23	pad	2
D-cycloserine treatment (*ΔmepM*)	S2E	KS81	LB+ 1 mM D-cycloserine	NA	2	NA	0 (a)	0	NA	0.257	15.8	4.05	1.09	29	pad	2
Single cell growth without drug (*ΔmepM*)	S2E	KS81	LB	NA	2	NA	0 (a)	NA	NA	0.267	15	4	1.1	17	pad	2
D-cycloserine treatment (*ΔmepH*)	S2E	KS82	LB+ 1 mM D-cycloserine	NA	2	NA	0 (a)	0	NA	0.266	18.3	4.85	0.91	17	pad	2
Single cell growth without drug (*ΔmepH*)	S2E	KS82	LB	NA	2	NA	0 (a)	NA	NA	0.3	16	4.8	0.92	27	pad	2
Bending without drug (control)	3C, S3B	S290	RDM+glu	NA	0.5	NA	5 ‡	NA	NA	-	-	-	-	10	donut	3
Bending during D-cycloserine treatment	3C, S3B	S290	RDM+glu + 1 mM D-cycloserine	+ 0.5 M NaCl (final concentration)	0.5	NA	5 ‡	0	10	-	-	-	-	71	donut	3
Straightening without drug (control)	3E, S3C	S290	RDM+glu	NA	2	NA	0 (s)	NA	NA	-	-	-	-	24	donut → square *	2
Straightening during D-cycloserine treatmen	3E, S3C	S290	RDM+glu	+ 1 mM D-cycloserine	2	NA	0 (s)	0	10	-	-	-	-	29	donut → square *	3
Cell-width change during D-cycloserine treatment (control)	4, S4	S257	LB + 5 mM D-cycloserine	NA	2	0.5	-15 (s)	-15	-1	-	-	-	0.96	37	pad (h = 1 mm)	2
Cell-width change on hypo-osmotic ramp (control) ci = mOsm, cf = mOsm	4, S4	S257	LB (970 mOsm)	LB (145 mOsm)	2	0.5	-15 (s)	NA	NA	-	-	-	0.94	42	pad ** h_1= 1 mm, h_2= 3 mm	2
Cell-width change on hypo-osmotic ramp during D-cycloserine	4, S4	S257	LB + 5 mM D-cycloserine (970 mOsm)	LB + 5 mM D-cycloserine (145 mOsm)	2	0.5	-15 (s)	-15	-1	-	-	-	0.94	44	pad ** h_1= 1 mm, h_2= 3 mm	2

Columns: Δt**:** interval between images; σ: standard deviation of the Gauss filter. t_{exp}**:** time point of placing cells on the microscope support relative to time indicated in figure axis. t_{exp} = 0 min indicates placement of pad onto cells. If indicated, cell division was inhibited by *sulA* induction (s) or 10 μg/mL aztreonam (a) since t_{exp} . t_{drug}**:** time of drug addition relative to time indicated in figure axis. t_{MreB}**:** time of MreB arrest relative to time indicated in figure axis. Please note that the difference of measurements of growth as a function of time after MreB-motion arrest (Fig. 1 and Fig. S1) vs measurements of MreB motion as a function of time after drug treatment (Table S2). ρ **,** S/M **,** S/V**,** W are the values at t = 0 min (Figs S1B-C, S1E, 2B) or the values of the initial timepoint (Figs. S2D-E) or otherwise the values used for normalization. n_{cells}**:** number of considered cells. **support:** microscopy support (agarose pad: 'pad', sticky-Slide I Luer (ibidi): 'flow chamber', donut-shaped chamber: 'donut', squared agarose-based chamber: 'square'). **biological replicates:** Number of repeated experiments from independent culture starting from separate colonies.

NA: Not applicable

¶: prior to microscopy, cells were grown in LB + 0.2% glucose, then washed and placed on an agarose pad containing Medium #1.

‡: Prior to t_{exp} (time when cells were loaded to donuts), *sulA* induction and D-cycloserine treatment were started in liquid culture at t = -10 min, 0 min respectively.

†: time when drug was added as a droplet to agarose pads, meaning drug arrival is gradual.

††: Medium #2 was added at t = -9 min. Glucose 1% was added at t = 36 min.

* cells were first bent in the donut chamber with Medium #1, subsequently they were transferred to the squared chamber with Medium #2 at t = 0.

** first pad with thickness h1 and Medium #1 covered by second pad with thickness h2 and Medium #2 at time t = 0. ci and cf are initial and calculated final concentration after equilibration of NaCl.

§ replicate in rich medium for Fig. 1B

Appendix Table S2: Detailed information of MreB experiment by fluorescence microscopy.

This table includes experimental types, corresponding figures and movies, used strains, growth media, time of MreB arrest, cell numbers, and replicates. . See description of columns below the table.

Experiment	Figure/Movie	Strain	Medium #1	Medium #2	Δt (sec)	duration (sec)	t_{exp} (min, method)	t_{drug} (min)	t_{arrest} (min)	n_{cells}	support	biological replicates
D-cycloserine treatment	1A, Movie EV1	S257	MM+glu	+ D-cycloserine 1 mM	1	60	-15 (s)	0	21	15	flow chamber	2 + Fig. S1D for RDM+glu
foscomycin treatment	1A, Movie EV2	S257	MM+glu	+ foscomycin 500 µg/mL	1	60	-15 (s)	0	30	13	flow chamber	2 + Fig. S1D for RDM+glu
vancomycin treatment	1A, Movie EV3	S382	MM+glu	+ vancomycin 100 µg/mL	1	60	-15 (s)	0	2	11	flow chamber	2 + Fig. S1D + Movie EV7 for RDM+glu
D-cycloserine treatment	S1D	S257	RDM+glu	+ D-cycloserine 1 mM	2	20	-9 (s)	0†	13	3	pad	§
foscomycin treatment	S1D	S257	RDM+glu	+ foscomycin 500 µg/mL	2	20	-10 (s)	0†	15	7	pad	§
vancomycin treatment	S1D	S382	RDM+glu	+ vancomycin 100 µg/mL	2	30	-6 (a)	0†	5	8	pad	§
MreB and single cell timelapse during	Movie EV7	S382	RDM+glu	+ vancomycin 100 µg/mL	20	1800	-8 (s)	-7	0	NA	pad	§
Complex nutrient shift during vancomycin	Movie EV11	S382	MM+glu	+ vancomycin 100 µg/mL, 0.5% αMG, 0.25% 2DG	2	20	-49 (s)	0	9	NA	pad	2
D-cycloserine treatment	Movie EV13	b183	LB+ D-cycloserine 1 mM	NA	1	60	0 (a)	0	11	NA	pad	2
D-cycloserine treatment	Movie EV14	S257	RDM+glu + D-cycloserine 1 mM	NA	1	60	0 (s)	0	10	NA	pad	2
D-cycloserine treatment	Movie EV17	S257	LB + 5 mM D-cycloserine (970 mOsm)	NA	1	60	0 (s)	0	14	NA	pad	2

Columns: Δt**:** interval between images; σ: standard deviation of the Gauss filter. t_{exp}**:** time point of placing cells on the microscope support relative to time indicated in figure axis. t_{exp} = 0 min indicates placement of pad onto cells. t_{drug}**:** time of drug addition relative to time indicated in figure axis. t_{arrest}**:** time of MreB-motion stop related to time indicated in the corresponding figures or movies. n_{cells}**:** average number of cells analyzed for MreB activity per timepoint. **support:** microscopy support (agarose pad: 'pad', sticky-Slide I Luer (ibidi): 'flow chamber'. **biological replicates:** Number of repeated experiments from independent

NA: Not applicable

†: time when drug was added as a droplet to agarose pads, meaning drug arrival is grac

§ replicate in rich medium for Fig. 1A

Appendix Table S3: Plasmid list

Plasmid	Genotype	Source
pCP20	*FLP*+, λ ci857+, λ p^R Rep^{ts}, Ap^R, Cm^R	(Cherepanov & Wackernagel, 1995)
pDB192	*bla* P_{lac}::*sulA*	Gift from Jun lab (UCSD) (de Boer *et al*, 1990)
pBAD30	Expression vector with P_{BAD} promoter	(Guzman *et al*, 1995)
pBC04	P_{BAD}-*mepS*	This study
pKY01	P_{BAD}-*mepS*^{C68A}	This study

Appendix Table S4: Strain list

Strain	Genotype	Construction
E. coli MG1655	wildtype	Gift from Ghigo lab (Institut Pasteur, Paris)
E. coli S290	MG1655/pDB192	(Oldewurtel *et al*, 2021)
E. coli S458	*lysA::kan*/pDB192	MG1655← P1(Keio Δ*lysA*)*, pDB192
E. coli NO34	*mreB::mreB-msfGFPsw, kan*	(Ouzounov *et al*, 2016)
E. coli NO53	*mreB*::*mreB* msfGFPsw	(Ouzounov *et al*, 2016)
E. coli S257	*mreB*::*mreB* msfGFPsw/pDB192	NO53←pDB192
E. coli NR693	MC4100 *lptD* 4213, *carB*::*Tn10*	(Ruiz *et al*, 2005)
E. coli EB03	*lptD* 4213, *carB*::*Tn10*	MG1655←P1(NR693)
E. coli EB06	*lptD* 4213	EB03←P1(MG1655)
E. coli S380	*lptD* 4213/pDB192	EB06←pDB192
E. coli S381	*lptD* 4213, *lysA::kan*/pDB192	S380←P1(Keio Δ*lysA*) *
E. coli S382	*lptD* 4213, *mreB*::*mreB-msfGFPsw kan*/pDB192	S380←P1(NO34)
E. coli EL162	*mepS*::*kan*	MG1655→P1(Keio Δ*mepS*) *
E. coli EL54	Δ*mepS*	EL162←pCP20
E. coli b42	Δ*mepS*/pBC04	MG1655←pBC04
E. coli b55	Δ*mepS*/pBAD30	EL54←pBAD30
E. coli S606	Δ*mepS*/pKY01	EL54←pKY01
E. coli KS81	*mepM*::*kan*	MG1655←P1 (Keio Δ*mepM*) *
E. coli KS82	*mepH*::*kan*	MG1655←P1 (Keio Δ*mepH*) *
S. enterica serovar Typhimurium SL1344	Δ*invA* Δ*sseB*	(Crouse *et al*, 2020)
V. cholerae E7946	wildtype	Gift from Doerr lab (Cornell)

* Keio single-gene deletion mutants are described in (Baba *et al*, 2006).

Appendix Table S5: Primer list

Primer name	Sequence (5' to 3')
P001	TGACTGACGAGCTCAGGAGGAATTCACCATGGTCAAATCTCAACCGATTTTG
P002	GTCAGTCATCTAGATTAGCTGCGGCTGAGAACCCG
P003	GCACTAAAAAAGGTATCGATGCTTCTGGTTTCGTACAGCGTAC
P004	CGTAATTTTTTTAAGGCAGTTATTGGTGCCCTTAAACG
P005	GTACGCTGTACGAAACCAGAAGCATCGATACCTTTTTTAGTGC
P006	CGTTTAAGGGCACCAATAACTGCCTTAAAAAAATTACG

Supplementary References

de Boer PA, Crossley RE & Rothfield LI (1990) Central role for the Escherichia coli minC gene product in two different cell division-inhibition systems. *Proc Natl Acad Sci U S A* 87: 1129–1133

Cherepanov PP & Wackernagel W (1995) Gene disruption in Escherichia coli: TcR and KmR cassettes with the option of Flp-catalyzed excision of the antibiotic-resistance determinant. *Gene* 158: 9–14

Crouse A, Schramm C, Emond-Rheault J-G, Herod A, Kerhoas M, Rohde J, Gruenheid S, Kukavica-Ibrulj I, Boyle B, Greenwood CMT, *et al* (2020) Combining Whole-Genome Sequencing and Multimodel Phenotyping To Identify Genetic Predictors of *Salmonella* Virulence. *mSphere* 5: e00293-20

Guzman LM, Belin D, Carson MJ & Beckwith J (1995) Tight regulation, modulation, and high-level expression by vectors containing the arabinose PBAD promoter. *Journal of Bacteriology* 177: 4121–4130

Oldewurtel ER, Kitahara Y & van Teeffelen S (2021) Robust surface-to-mass coupling and turgor-dependent cell width determine bacterial dry-mass density. *Proc Natl Acad Sci U S A* 118: e2021416118

Ouzounov N, Nguyen JP, Bratton BP, Jacobowitz D, Gitai Z & Shaevitz JW (2016) MreB Orientation Correlates with Cell Diameter in Escherichia coli. *Biophysical Journal* 111: 1035–1043

Ruiz N, Falcone B, Kahne D & Silhavy TJ (2005) Chemical conditionality: a genetic strategy to probe organelle assembly. *Cell* 121: 307–317

Supplementary Figures

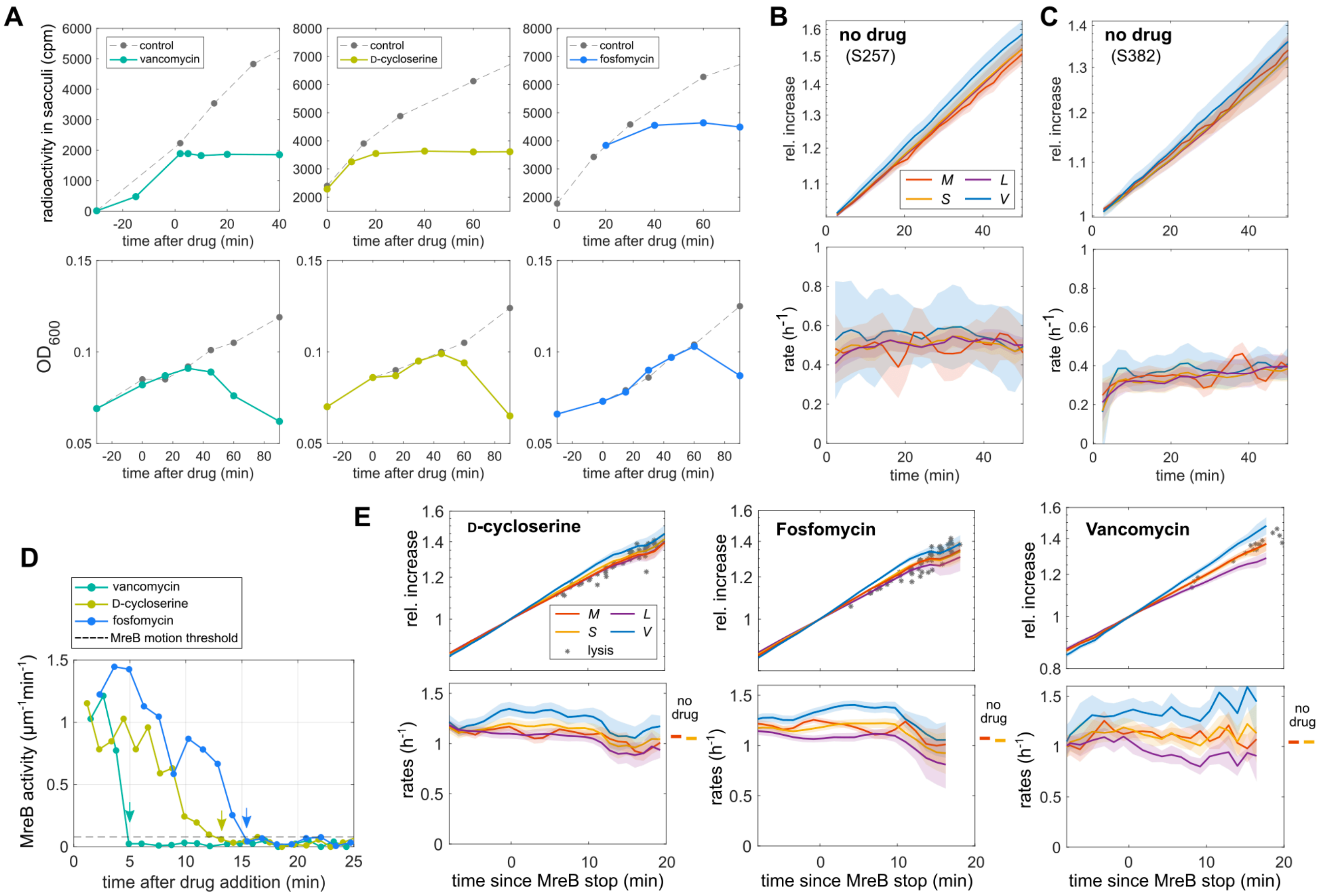

Appendix Figure S1. Confirmation of cell wall synthesis arrest by radiolabeling experiments and cell wall-synthesis independent growth in rich medium.

A: Increase of radioactivity in isolated cell wall sacculi after addition of ^{3}H-mDAP and OD measurements in the absence or in the presence of drug. S458 (*lysA*::kan/pDB192) and S381 (*lptD* 4213, *lysA*::kan/pDB192) cells were grown in MM+glu supplemented with 0.4 mM lysine and ^{3}H-mDAP was added to the culture at t = -30 min. D-cycloserine (1 mM) and fosfomycin (500 µg/mL) were added to S458 culture, and vancomycin (100 µg/mL) was added to S381 culture at t = 0 min. ^{3}H-mDAP incorporation stops after <2 min (vancomycin), ~10-20 min (D-cycloserine), and ~20-40 min (fosfomycin). OD600 of the same culture but without ^{3}H-mDAP was measured during drug treatments. These experiments were repeated twice from a different colony.

B-C: Single-cell timelapse of S257 (B) and S382 (C) cells in MM+glu without drug in a flow chamber. Relative increase (top) and growth rates (bottom) of volume, mass, surface and length.

D-E: MreB activity and single-cell growth after cell wall-synthesis inhibition in rich medium (RDM+glu), in analogy to experiments in minimal medium (Fig. 1A-B). Different from Fig. 1A-B, cells were immobilized under agarose pads, and D-cycloserine (1 mM), fosfomycin (500 µg/ml), or vancomycin (100 µg/ml) were added in form of droplets on top of the pads. Drugs thus reach the cells by diffusion within minutes (~8 min for 1/10 final concentration in the case of vancomycin). Strains used were S257 (D-cycloserine, fosfomycin) or S382 (vancomycin).

Solid lines + shadings = average ± 2*standard error. For details including absolute values of relative quantities, cell numbers, and replicates see Tables S1-2.

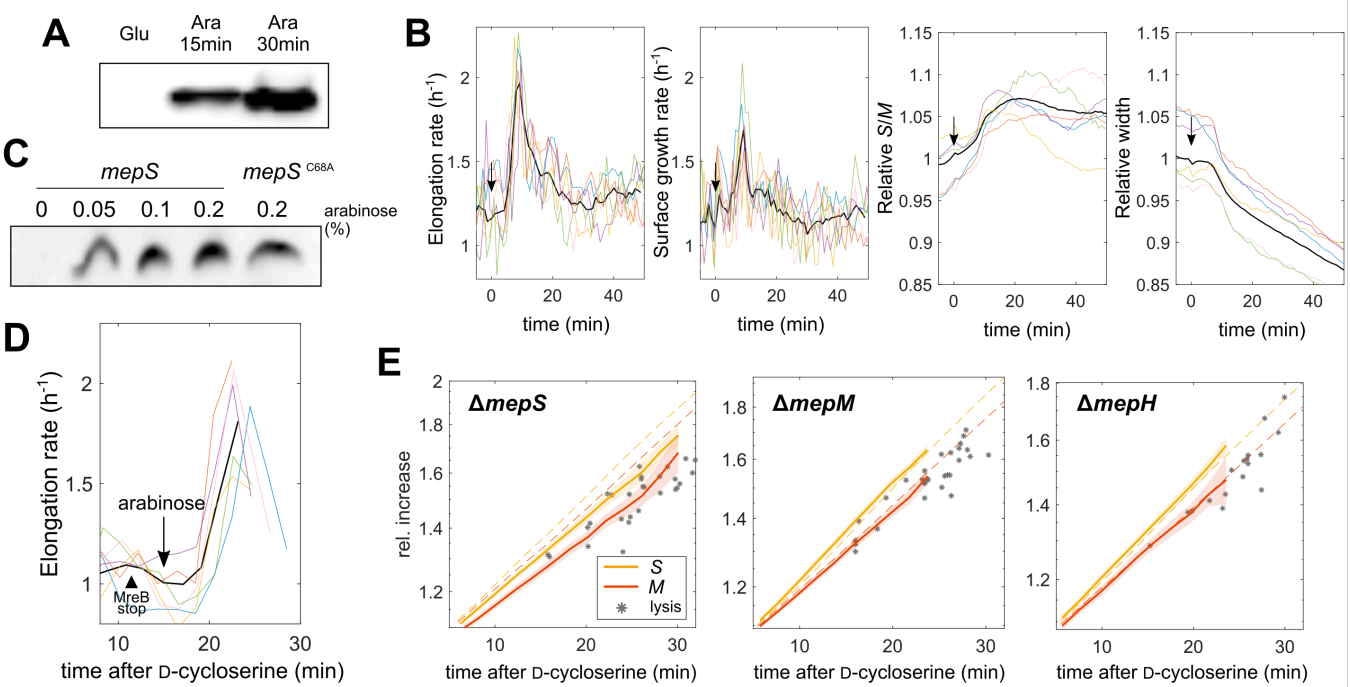

Appendix Figure S2. MepS overexpression transiently accelerates cell elongation independently of cell wall synthesis.

A: MepS protein levels in the membrane fraction during growth of strain b42 (Δ*mepS* pBAD30-*mepS*) in LB+glu (0.2%) and after washing + arabinose induction (0.2%; *t* = 15 min and 30 min after induction) using Western blot. This experiment was repeated twice from a different colony.

B: Single cell traces during MepS overexpression using strain b42 (Δ*mepS* pBAD30-*mepS*) as a function of time since induction, corresponding to Fig. 2A. From left to right: Rates of elongation and surface growth; relative change of the surface-to-mass ratio and width normalized by the average values at *t* = 0 min. Black bold line indicates the average shown in Fig. 2.

C: MepS protein levels in the membrane fraction during growth of strains b42 (Δ*mepS* pBAD30-*mepS*) or S606 (Δ*mepS* pBAD30-*mepS*^{C68A}) in LB measured 20 min after induction (0.05-0.2% arabinose) using Western blot. This experiment was repeated twice from a different colony.

D: Single-cell elongation rate during MepS overexpression of strain b183 (Δ*mepS mreB-msfGFP* pBAD30-*mepS*) as a function of time after placing cells on agarose pad containing D-cycloserine (1 mM) (thin lines: single cells; thick black line: average) shows equal burst as MepS induction in non-drug-treated cells (Fig. S2B). MepS was induced 15 minutes after D-cycloserine treatment (0.2% arabinose as a droplet on top of agarose pad), well after observation of MreB-motion stop in a parallel experiment without MepS induction (11 min after drug treatment) (Movie EV13).

E: Single-cell growth of endopeptidase mutants EL54 (Δ*mepS*), KS81 (Δ*mepM*) and KS82 (Δ*mepH*) grown in LB+D-cycloserine (1 mM) as a function of time after placing cells on agarose pad containing the drug. Relative increase of surface and mass. Gray asterisks indicate single-cell mass at lysis and dashed lines indicate averages in the absence of drug. Solid lines + shadings = average ± 2*standard error.

For details including absolute values of relative quantities, cell numbers, and replicates see Table S1.

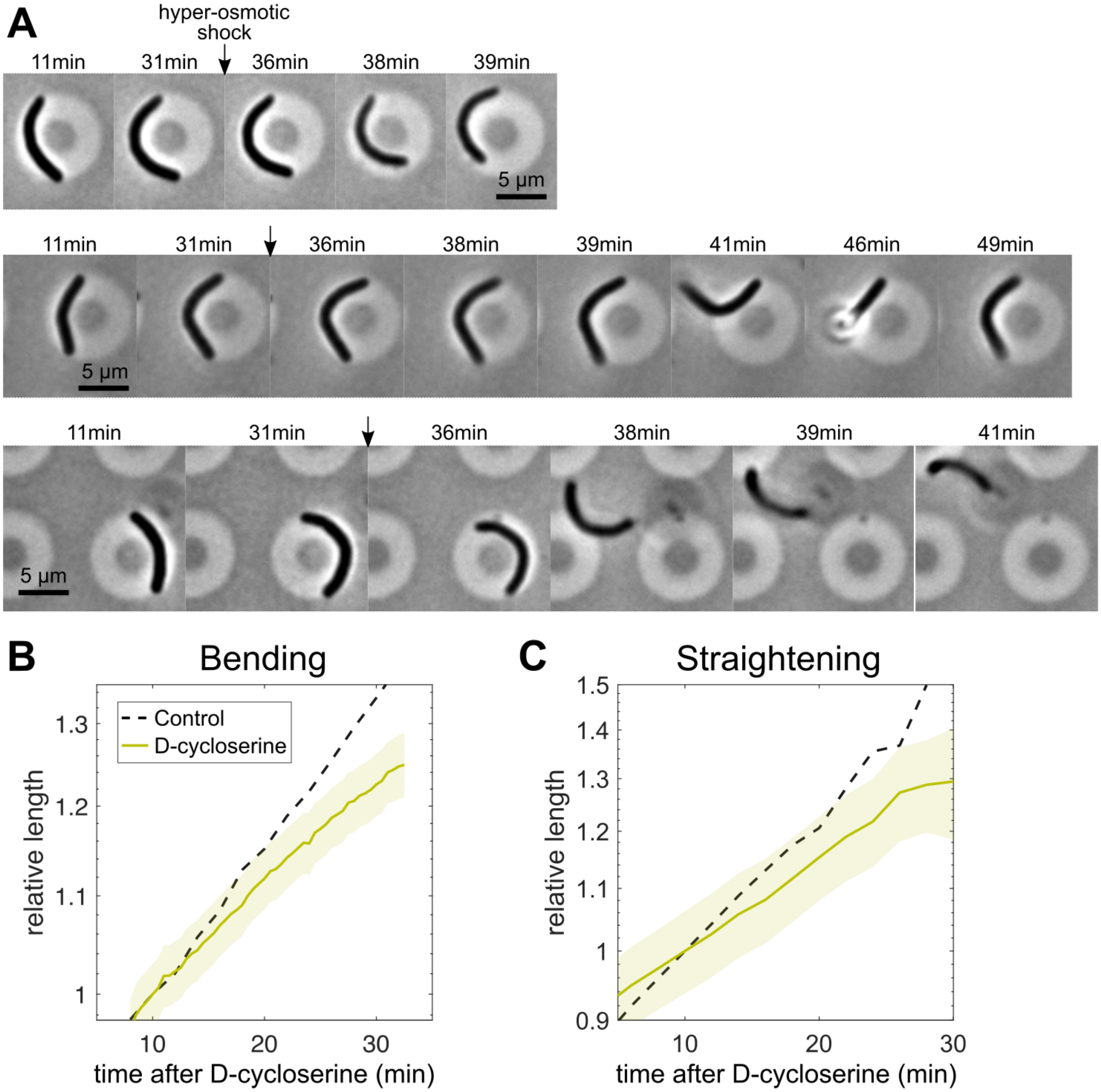

Appendix Figure S3. Bending and straightening cells during cell wall-synthesis inhibition.

A: Representative single cells bending in donut microchambers in the presence of 1 mM D-cycloserine as in Fig. 3B. Arrows indicate hyper-osmotic shock as in Fig. 3B.

B: Relative length of single cells bending in the donut microchambers in the presence or absence of 1 mM D-cycloserine corresponding to Fig. 3B-C. (here and in C, solid lines + shadings = average ± 2*standard error)

C: Relative length of single cells straightening in the presence or absence of D-cycloserine after release from donut microchambers corresponding to Fig. 3D-E.

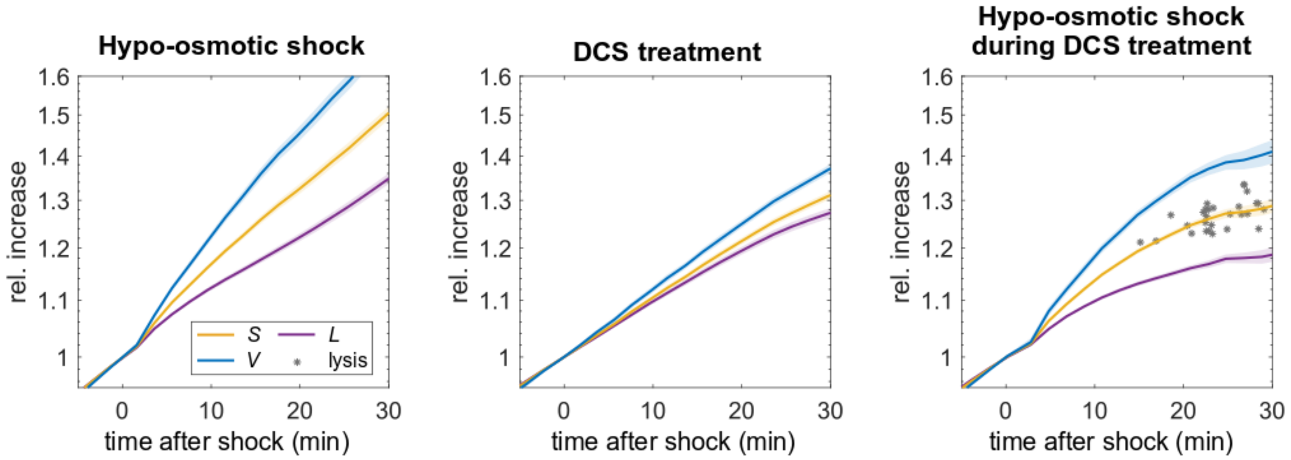

Appendix Figure S4. Hypo-osmotic ramp and D-cycloserine treatment.

Relative increase of surface, volume, and length during hypo-osmotic ramp (left), D-cycloserine treatment (middle), and combined hypo-osmotic ramp + D-cycloserine treatment (right). Conditions are described in Fig. 4. Gray asterisks indicate single-cell surface area at lysis. (solid lines + shadings = average ± 2*standard error)
