# Supplementary material for: Cell envelope growth of Gram‐negative bacteria proceeds independently of cell wall synthesis
Source: EMBO J. 2023 Jun 1;42(14):e112168. doi: 10.15252/embj.2022112168 (PMC10350831; doi:10.15252/embj.2022112168)
Supplement: Supplementary file 4 — Movie EV3 [file EMBJ-42-e112168-s017.zip › EMBOJ-2022-112168_MovieEV3/caption.docx]

**Movie EV3. MreB-msfGFP motion during inhibition of cell-wall insertion in minimal medium corresponding to Fig. 1A**. MreB-msfGFP motion in cells growing in flow chambers (MM+glu) during treatment with vancomycin. Each panel shows a 60 s-long movie started at different time points after drug exposure as indicated in the movies and in Fig. 1A (2 min). Strain S382 was used.
