# Supplementary material for: Cell envelope growth of Gram‐negative bacteria proceeds independently of cell wall synthesis
Source: EMBO J. 2023 Jun 1;42(14):e112168. doi: 10.15252/embj.2022112168 (PMC10350831; doi:10.15252/embj.2022112168)
Supplement: Supplementary file 5 — Movie EV4 [file EMBJ-42-e112168-s019.zip › EMBOJ-2022-112168_MovieEV4/caption.docx]

**Movie EV4: Single-cell growth upon inhibition of cell-wall synthesis in minimal medium corresponding to Fig. 1B.** Timelapses of single-cell growth (phase-contrast microscopy) in flow chambers (MM+glu) during treatment with D-cycloserine. Time stamps indicate time with respect to MreB-motion arrest shown in Fig 1A. Strain S257 was used.
