# Supplementary material for: Cell envelope growth of Gram‐negative bacteria proceeds independently of cell wall synthesis
Source: EMBO J. 2023 Jun 1;42(14):e112168. doi: 10.15252/embj.2022112168 (PMC10350831; doi:10.15252/embj.2022112168)
Supplement: Supplementary file 8 — Movie EV7 [file EMBJ-42-e112168-s001.zip › EMBOJ-2022-112168_MovieEV7/caption.docx]

**Movie EV7: Single-cell growth and MreB-msfGFP motion within the same cell during vancomycin treatment in rich medium.** Simultaneous phase-contrast and fluorescence time-lapses of a S382 cell during vancomycin treatment on an agarose pad (RDM+glu). Time stamps indicate time with respect to MreB-motion arrest. The movie shows continuous size growth after MreB-motion stop in the same condition as Fig. S1E.
