# Supplementary material for: Cell envelope growth of Gram‐negative bacteria proceeds independently of cell wall synthesis
Source: EMBO J. 2023 Jun 1;42(14):e112168. doi: 10.15252/embj.2022112168 (PMC10350831; doi:10.15252/embj.2022112168)
Supplement: Supplementary file 9 — Movie EV8 [file EMBJ-42-e112168-s004.zip › EMBOJ-2022-112168_MovieEV8/caption.docx]

**Movie EV8: Single-cell growth during inhibition of cell-wall synthesis in *S. enterica* Serovar Typhimurium corresponding to Fig. 1C.** Single-cell time lapse during D-cycloserine treatment on an agarose pad (RDM). Time stamps indicate time with respect to the time when cells were put on the agarose pad containing D-cycloserine (= time after the drug treatment started).
