# Supplementary material for: Cell envelope growth of Gram‐negative bacteria proceeds independently of cell wall synthesis
Source: EMBO J. 2023 Jun 1;42(14):e112168. doi: 10.15252/embj.2022112168 (PMC10350831; doi:10.15252/embj.2022112168)
Supplement: Supplementary file 11 — Movie EV10 [file EMBJ-42-e112168-s016.zip › EMBOJ-2022-112168_MovieEV10/caption.docx]

**Movie EV10: Single-cell growth during complex nutrient shift (vancomycin + glucose analogs) corresponding to Fig. 1D.** Phase-contrast movie of S382 cell grown under agarose pad, as a function of time after MreB-motion stop (see Movie EV11).
