# Supplementary material for: Cell envelope growth of Gram‐negative bacteria proceeds independently of cell wall synthesis
Source: EMBO J. 2023 Jun 1;42(14):e112168. doi: 10.15252/embj.2022112168 (PMC10350831; doi:10.15252/embj.2022112168)
Supplement: Supplementary file 12 — Movie EV11 [file EMBJ-42-e112168-s020.zip › EMBOJ-2022-112168_MovieEV11/caption.docx]

**Movie EV11: MreB motion during complex nutrient shift (vancomycin + glucose analogs) corresponding to Fig. 1D.** MreB-msfGFP motion of S382 cells obtained from the same experiment shown in Movie EV10 and Fig. 1D. Each panel shows a 20 s-long movie started before and after drug exposure as indicated. MreB motion stops 9 min after drug treatment.
