# Supplementary material for: Cell envelope growth of Gram‐negative bacteria proceeds independently of cell wall synthesis
Source: EMBO J. 2023 Jun 1;42(14):e112168. doi: 10.15252/embj.2022112168 (PMC10350831; doi:10.15252/embj.2022112168)
Supplement: Supplementary file 13 — Movie EV12 [file EMBJ-42-e112168-s003.zip › EMBOJ-2022-112168_MovieEV12/caption.docx]

**Movie EV12: Single-cell growth during DD-endopeptidase MepS overexpression corresponding to Fig. 2A.** Single-cell time lapse of a b42 cell during MepS overexpression on an agarose pad (LB). Time stamps are relative to time of arabinose (inducer) addition onto the pad in form of a droplet.
