# Supplementary material for: Cell envelope growth of Gram‐negative bacteria proceeds independently of cell wall synthesis
Source: EMBO J. 2023 Jun 1;42(14):e112168. doi: 10.15252/embj.2022112168 (PMC10350831; doi:10.15252/embj.2022112168)
Supplement: Supplementary file 14 — Movie EV13 [file EMBJ-42-e112168-s014.zip › EMBOJ-2022-112168_MovieEV13/caption.docx]

**Movie EV13: MreB-msfGFP motion during inhibition of cell-wall synthesis in LB corresponding to Fig. S2D.** MreB-msfGFP motion in b183 cells during D-cycloserine treatment on a LB agarose pad (analogous to Fig. S2D, but without addition of arabinose). Each panel shows a 60 s-long movie started at different time points with respect to the time when cells were put on the agarose pad containing D-cycloserine. MreB motion stops after 11 min of drug treatment.
