# Supplementary material for: Cell envelope growth of Gram‐negative bacteria proceeds independently of cell wall synthesis
Source: EMBO J. 2023 Jun 1;42(14):e112168. doi: 10.15252/embj.2022112168 (PMC10350831; doi:10.15252/embj.2022112168)
Supplement: Supplementary file 15 — Movie EV14 [file EMBJ-42-e112168-s015.zip › EMBOJ-2022-112168_MovieEV14/caption.docx]

**Movie EV14: MreB-msfGFP motion during D-cycloserine treatment mimicking conditions of bending (Fig. 3B-C) and straightening (Fig. 3D-E) experiments.** Movie of MreB-msfGFP in S257 cells growing on agarose pads (RDM+glu) containing 1 mM D-cycloserine. Each panel shows a 60 s-long movie started at different time points after drug treatment (= time when cells were put on the agarose pad containing D-cycloserine). MreB motion stops 10 min after drug treatment.
