# Supplementary material for: Cell envelope growth of Gram‐negative bacteria proceeds independently of cell wall synthesis
Source: EMBO J. 2023 Jun 1;42(14):e112168. doi: 10.15252/embj.2022112168 (PMC10350831; doi:10.15252/embj.2022112168)
Supplement: Supplementary file 16 — Movie EV15 [file EMBJ-42-e112168-s012.zip › EMBOJ-2022-112168_MovieEV15/caption.docx]

**Movie EV15: Bending cells in the absence of cell-wall synthesis corresponding to Fig 3B-C.** Single-cell time lapse of a D-cycloserine treated S290 cell in a donut chamber (RDM+glu). Time stamps relative to time of treatment with D-cycloserine. Cells were loaded into donuts at time point 8 min, meaning cells were briefly pre-exposed to drug prior to loading.
