# Supplementary material for: Cell envelope growth of Gram‐negative bacteria proceeds independently of cell wall synthesis
Source: EMBO J. 2023 Jun 1;42(14):e112168. doi: 10.15252/embj.2022112168 (PMC10350831; doi:10.15252/embj.2022112168)
Supplement: Supplementary file 17 — Movie EV16 [file EMBJ-42-e112168-s010.zip › EMBOJ-2022-112168_MovieEV16/caption.docx]

**Movie EV16: Straightening cells in the absence of cell-wall synthesis corresponding to Fig. 3D-E.** Single-cell time lapse of a D-cycloserine treated S290 cell in a squared agarose-based chamber. Time stamps relative to time of cell loading to squared chambers from donut microchambers (= time of start of D-cycloserine treatment).
