# Supplementary material for: Cell envelope growth of Gram‐negative bacteria proceeds independently of cell wall synthesis
Source: EMBO J. 2023 Jun 1;42(14):e112168. doi: 10.15252/embj.2022112168 (PMC10350831; doi:10.15252/embj.2022112168)
Supplement: Supplementary file 18 — Movie EV17 [file EMBJ-42-e112168-s002.zip › EMBOJ-2022-112168_MovieEV17/caption.docx]

**Movie EV17: MreB-msfGFP motion during D-cycloserine treatment under high salt condition corresponding to conditions in Fig. 4.** MreB-msfGFP motion in S257 cells growing on a LB agarose pad (970 mOsm adjusted by NaCl) during treatment with 5 mM D-cycloserine (the same condition as Fig. 4, but independent experiment). Each panel shows a 60 s-long movie started at different time points after drug treatment (= time when cells were put on the agarose pad containing D-cycloserine). MreB motion stops 14 min after drug treatment.
