# Supplementary material for: Cell envelope growth of Gram‐negative bacteria proceeds independently of cell wall synthesis
Source: EMBO J. 2023 Jun 1;42(14):e112168. doi: 10.15252/embj.2022112168 (PMC10350831; doi:10.15252/embj.2022112168)
Supplement: Supplementary file 19 — Movie EV18 [file EMBJ-42-e112168-s007.zip › EMBOJ-2022-112168_MovieEV18/caption.docx]

**Movie EV18: Single-cell growth during D-cycloserine treatment under high salt condition corresponding to Fig. 4.** Single-cell time lapse of a S257 cells grown on an agarose pad containing D-cycloserine (5 mM) for 15 min prior to a hypo-osmotic ramp at t = 0 min.
