# Supplementary material for: Cell envelope growth of Gram‐negative bacteria proceeds independently of cell wall synthesis
Source: EMBO J. 2023 Jun 1;42(14):e112168. doi: 10.15252/embj.2022112168 (PMC10350831; doi:10.15252/embj.2022112168)
Supplement: Supplementary file 20 [file EMBJ-42-e112168-s018.pdf]

THE EMBO JOURNAL

Cell envelope growth of Gram-negative bacteria proceeds independently of cell wall synthesis

Enno Oldewurtel, Yuki Kitahara, Baptiste Cordier, Richard Wheeler, Gizem Ozbaykal, Elisa Brambilla, Ivo GOMPERTS BONECA, Lars Renner, and Sven van Teeffelen
DOI: 10.15252/embj.2022112168

Corresponding author(s): Sven van Teeffelen (sven.vanteeffelen@gmail.com) , Lars Renner (lars.d.renner@gmail.com)

Review Timeline:

Presubmission Date:	20th Jul 22
Presubmission Editorial Decision:	21st Jul 22
Revision Received:	25th Jul 22
Editorial Decision:	30th Aug 22
Revision Received:	19th Mar 23
Editorial Decision:	5th May 23
Revision Received:	11th May 23
Accepted:	16th May 23

Editor: Ieva Gailite

Transaction Report:

(Note: With the exception of the correction of typographical or spelling errors that could be a source of ambiguity, letters and reports are not edited. Depending on transfer agreements, referee reports obtained elsewhere may or may not be included in this compilation. Referee reports are anonymous unless the Referee chooses to sign their reports.)

Presubmission Editorial Decision **21st Jul 2022**

Dear Sven,

Thanks very much for contacting me to enquire about the suitability of your manuscript for The EMBO Journal. I have now looked into your study, and I would like to send it out for full peer review. You can use the link below to initiate full submission of the manuscript. I look forward to receiving your submission!

With best regards,

Ieva

Ieva Gailite, PhD
Scientific Editor
The EMBO Journal
Meyerhofstrasse 1
D-69117 Heidelberg
Tel: +4962218891309
i.gailite@embojournal.org

To submit your manuscript, please follow this link.

Link Not Available

Please do not share this URL as it will give anyone who clicks it access to your account.

Please note that for technical reasons, this link will take you to a page for uploading revised manuscripts - once we receive the submission, it will be formally turned into an original submission.

1st Editorial Decision 30th Aug 2022

Dear Sven,

Thank you for submitting your manuscript for consideration by the EMBO Journal. I sincerely apologise for the protracted assessment of your manuscript due to delayed submission of referee reports. We have now received comments from three reviewers, which are included below for your information.

As you will see from the reports, the reviewers find the described peptidoglycan synthesis independent regulation of cell wall expansion in E. coli of interest. However, in particular reviewer #2 finds that more stringent evidence for full inhibition of PG incorporation and the PG biosynthesis-independent function of MepS function in cell wall expansion from would be needed to support the conclusions. Furthermore, both reviewers #2 and #3 indicate that analysis of D,D-endopeptidase mutants would be needed, and question the proposed mechanism of MepS activity inhibition.

If you find that you are able to address the main issues raised by the reviewers, I would be happy to consider a revised version of the manuscript. I think it would be helpful to discuss the revision in more detail via email or phone/videoconferencing - please let me know which option you prefer. I should also add that it is The EMBO Journal policy to allow only a single major round of revision and that it is therefore important to resolve the main concerns at this stage.

We generally allow three months as standard revision time. As a matter of policy, competing manuscripts published during this period will not negatively impact on our assessment of the conceptual advance presented by your study. However, please contact me as soon as possible upon publication of any related work to discuss the appropriate course of action. Should you foresee a problem in meeting this three-month deadline, please contact us to arrange an extension.

When preparing your letter of response to the referees' comments, please bear in mind that this will form part of the Review Process File and will therefore be available online to the community. For more details on our Transparent Editorial Process, please visit our website: https://www.embopress.org/page/journal/14602075/authorguide#transparentprocess. Please also see the attached instructions for further guidelines on preparation of the revised manuscript.

Please feel free to contact me if you have any further questions regarding the revision. Thank you for the opportunity to consider your work for publication, and I look forward to discussing your revision.

With best regards,

Ieva

Ieva Gailite, PhD
Senior Scientific Editor
The EMBO Journal
Meyerhofstrasse 1
D-69117 Heidelberg
Tel: +4962218891309
i.gailite@embojournal.org

When submitting your revised manuscript, please carefully review the instructions below and include the following items:

1) a .docx formatted version of the manuscript text (including legends for main figures, EV figures and tables). Please make sure that the changes are highlighted to be clearly visible.

2) individual production quality figure files as .eps, .tif, .jpg (one file per figure).

3) a .docx formatted letter INCLUDING the reviewers' reports and your detailed point-by-point response to their comments. As part of the EMBO Press transparent editorial process, the point-by-point response is part of the Review Process File (RPF), which will be published alongside your paper.

4) a complete author checklist, which you can download from our author guidelines (https://wol-prod-cdn.literatumonline.com/pb-assets/embo-site/Author Checklist%20-%20EMBO%20J-1561436015657.xlsx). Please insert information in the checklist that is also reflected in the manuscript. The completed author checklist will also be part of the RPF.

5) Please note that all corresponding authors are required to supply an ORCID ID for their name upon submission of a revised

manuscript.

6) Before submitting your revision, primary datasets produced in this study need to be deposited in an appropriate public database (see https://www.embopress.org/page/journal/14602075/authorguide#datadeposition). Please remember to provide a reviewer password if the datasets are not yet public. Please note that the Data Availability Section is restricted to new primary data that are part of this study. If no data deposition in external databases is needed for this paper, please then state in this section: This study includes no data deposited in external repositories.
*** Note - All links should resolve to a page where the data can be accessed. ***

7) Our journal encourages inclusion of *data citations in the reference list* to directly cite datasets that were re-used and obtained from public databases. Data citations in the article text are distinct from normal bibliographical citations and should directly link to the database records from which the data can be accessed. In the main text, data citations are formatted as follows: "Data ref: Smith et al, 2001" or "Data ref: NCBI Sequence Read Archive PRJNA342805, 2017". In the Reference list, data citations must be labeled with "[DATASET]". A data reference must provide the database name, accession number/identifiers and a resolvable link to the landing page from which the data can be accessed at the end of the reference. Further instructions are available at .

8) We would also encourage you to include the source data for figure panels that show essential data. Numerical data can be provided as individual .xls or .csv files (including a tab describing the data). For 'blots' or microscopy, uncropped images should be submitted (using a zip archive or a single pdf per main figure if multiple images need to be supplied for one panel). Additional information on source data and instruction on how to label the files are available at .

9) We replaced Supplementary Information with Expanded View (EV) Figures and Tables that are collapsible/expandable online (see examples in https://www.embopress.org/doi/10.15252/embj.201695874). A maximum of 5 EV Figures can be typeset. EV Figures should be cited as 'Figure EV1, Figure EV2" etc. in the text and their respective legends should be included in the main text after the legends of regular figures.

- For the figures that you do NOT wish to display as Expanded View figures, they should be bundled together with their legends in a single PDF file called *Appendix*, which should start with a short Table of Content. Appendix figures should be referred to in the main text as: "Appendix Figure S1, Appendix Figure S2" etc. See detailed instructions regarding expanded view here: .

- Additional Tables/Datasets should be labelled and referred to as Table EV1, Dataset EV1, etc. Legends have to be provided in a separate tab in case of .xls files. Alternatively, the legend can be supplied as a separate text file (README) and zipped together with the Table/Dataset file.

10) When assembling figures, please refer to our figure preparation guideline in order to ensure proper formatting and readability in print as well as on screen:
http://bit.ly/EMBOPressFigurePreparationGuideline

Please remember: Digital image enhancement is acceptable practice, as long as it accurately represents the original data and conforms to community standards. If a figure has been subjected to significant electronic manipulation, this must be noted in the figure legend or in the 'Materials and Methods' section. The editors reserve the right to request original versions of figures and the original images that were used to assemble the figure.

Further information is available in our Guide For Authors: https://www.embopress.org/page/journal/14602075/authorguide

We realize that it is difficult to revise to a specific deadline. In the interest of protecting the conceptual advance provided by the work, we recommend a revision within 3 months (28th Nov 2022). Please discuss the revision progress ahead of this time with the editor if you require more time to complete the revisions.

Referee #1:

The study by Oldewurtel et al. addresses the relationship between mass increase and cell wall homeostasis in Gram-negative bacteria. The main conclusion is that mass increase is independent of cell wall synthesis, but instead likely relies on cell wall lytic enzymes. This is a very well-designed study that addresses a crucial unknown aspect of cell envelope biology and, as a particularly exciting aspect of it, dispenses with the persistent dogma that cell wall synthesis rules all during envelope expansion. The conclusions are overall supported by the data and the appropriate controls are in place. I have only minor concerns.

Overall, while the main conclusion (PG synthesis is not required for envelope expansion) is well-supported, the evidence for autolysin involvement is indirect. Autolysin involvement logically follows from their PG inhibition experiments, but is not directly demonstrated (except for MepS overexpression, but this is in a different context than the main experimental system); conclusions regarding autolysin should thus be slightly softened (e.g., "can likely control..." in line 24

Fig. 1 would benefit from adding untreated cells as a comparison (panel 1B), as currently they only have mass growth in the presence of drugs. I think they might have these data, since they mention a slight disturbance in width homeostasis during drug treatment in the text (which presumably was compared to an untreated control). However, this is not necessary to support their main conclusion.

Other, very minor comments:

- Line 24: "Therefore" should be deleted - cell change in response to mechanical stress does not logically allow for the conclusion of the involvement of autolsysin. Perhaps replace with "overall, our data suggest..."; if the statement about autolysins includes the data mentioned earlier in the abstract, rather than just mechanical stress as implied by "therefore", it makes sense
- Line 34/35 - there is no evidence for this (though it is logical and reasonable to think so), so perhaps weaken to "are likely co-ordinated". Their own and other labs' data actually suggest that autolysin activity and at least aPBPs are not necessarily co-ordinated in a strict sense.
- Line 65 - "demonstrating" is a bit strong, perhaps change to "consistent with"
- Line 85: severely reduced or stopped? (though this is a minor rhetoric distinction) The figure suggests the latter.
- Line 103 needs a citation, e.g. PMID: 25884840
- Remove extraneous sentence in line 120/121
- Line 122 needs a citation, e.g. PMID: 23062283, and https://doi.org/10.1111/mmi.12059
- Line 139 - is this concentration/duration of treatment sufficient to stop MreB motion?
- Line 167/68 is a bit confusing, since "restoring pressure" implies an increase, while their cells actually are prevented from reducing turgor back to normal levels. Maybe replace with something like "restoring normal turgor" or similar?
- Line 285 - add more information on mass measurements. I know they refer to a previous paper, but it is easier on the reader if this procedure is explained again here.

Referee #2:

In this manuscript, the authors put forth an interesting hypothesis which challenges a long-held notion in bacterial physiology - that PG synthesis, or more specifically new PG insertion, is critical for cell expansion. Using extensive microscopy, authors study the rates of cell size, volume and surface increase upon inhibition of cell wall synthesis by targeting either PG precursor synthesis (fosfomycin, cycloserine) or PG polymerisation (vancomycin). Cessation of PG synthesis is verified by 3H-mDAP incorporation and reduction in MreB motion. They observe that cells continue to grow and elongate after inhibition of MreB motion, however the growth is transient and eventually cells lyse. Furthermore, such cell envelope expansion is perturbed synchronously with perturbation of biomass growth, mechanical strain and osmotic downshock.

Interestingly, the authors report that envelope growth is proportional to D,D-endopeptidase activity which raises the possibility of PG incorporation and hydrolysis being decoupled. The experiments are designed meticulously to study minute physical details of cell expansion on a short timescale.
However, the conclusions drawn here are overreaching, and the presented evidence is quite preliminary and does not sufficiently invalidate the need for PG insertion for cell envelope expansion. Several additional experiments are required to conclusively say that envelope growth does not require new PG insertion. It appears that cell envelope expansion, if truly independent of PG synthesis, is a transient event that is not physiologically sustainable as cells lyse shortly after PG synthesis inhibition.
Specific points are listed below -

1. The most crucial aspect of this study to be considered is - confirming whether there is a complete inhibition of PG incorporation. The evidence presented here has two caveats. Firstly, it is unclear from the description whether the point at which the growth monitoring starts (indicated as time since MreB stop in Fig. 1B) is the point of complete stoppage or significant reduction in MreB motion (described as 'severely reduced within 5-15 min depending on the drug' in line 85). At what time point in the movies S1-3 was growth monitoring initiated? Further, cessation of MreB motion is not indicative of absence of PG polymerisation, as aPBPs are known to work independently of the Rod complex (Cho 2016) and may still be sufficiently functional for PG incorporation here. Along the same lines, line 221 states 'Here, we found that the activity of aPBPs is not responsible for differential cell-envelope growth' - direct evidence for this has not been presented. Secondly, the point at which 3H-mDAP incorporation stops following drug treatment appears to be longer than the point at which MreB motion stops, which is where cell growth monitoring begins. Eg: It takes ~22 mins for mDAP incorporation to stop following fosfomycin treatment, although growth rate monitoring begins at ~15 mins post treatment (Figs 1A, 1B, S1A). In addition, the 3H-mDAP incorporation assay is performed in broth culture, while growth rate is monitored on agarose pad where diffusion time for the drug may lead to a further lag, because of which the time required for complete inhibition of PG incorporation may be underestimated here.

2. Fig. 1A, movie S1 shows that cell lysis directly coincides with the point of complete cessation of MreB motion following addition of D-cycloserine (~30 min). This further brings into question the duration for which cell envelope expansion proceeds after PG incorporation has completely stopped. This event appears to be transient and not physiologically sustainable as cells do not survive for long. Is the lysis due to turgor, or due to strict requirement of PG insertion for growth? If cell envelope growth is truly independent of PG incorporation, will the cells remain viable in hyper osmotic media where turgor will not lead to lysis?
3. Line 24 'cell-wall-cleaving enzymes can control envelope growth independently of synthesis' and Fig 2B, C - the experiment showing increase in cell size and volume upon overexpression of MepS does not include drug treatment for inhibition of PG incorporation. Therefore there is no direct evidence to state that the action of MepS is independent of PG synthesis.
4. Line 128 'elongation rate and surface rate increase only transiently (during 5-10 min), despite long-term enzyme overexpression, suggesting that envelope growth is likely not controlled through endopeptidase levels but through the regulation of activity at the single-enzyme level.'; Fig 2A; and lines 213-216 in discussion - was MepS expression checked over time? MepS half-life known to be very low and tightly regulated and may be reflected in the growth rate. A novel self-regulatory mechanism for MepS regulation, which has not been predicted by structural studies, need not be invoked based on this observation.
5. In similar lines, if one has to invoke the role of endopeptidases in insertion-independent envelope growth, then these experiments need to be repeated in appropriate mutants (lacking MepS or any other).
6. Line 96: The authors suggest that "cell-wall cleaving autolysins must be responsible for extending the area of the cell wall, independent of cell-wall insertion", since E. coli PG is mostly single-layered, how such process will allow PG extension without affecting PG integrity should be discussed. This is a point of particular interest as it can further explain the process of PG remodeling and re-structuring without new PG incorporation. Will endopeptidase deficient mutants fail in such process?
7. Line 119: Endopeptidase other than MepS can alter envelope growth? Because of the importance of the observation, as a control experiment MepS catalytic mutant should be used. More MepS may also titrate NlpI leading to reregulation of several hydrolases/ synthases (Banzhaf, 2020).
8. Lines 76-78: Logic of using SulA overexpression to inhibit FtsZ is not clear. None of the antibiotics used in this study mediate septation-induced lysis (Ref 26 talks about beta-lactam induced cell lysis).
9. Fig 3D - the duration of recovery (~20 mins) for both D-cycloserine and fosfomycin appears to be within the window between drug addition and cessation of mDAP incorporation. Therefore PG insertion playing a role in recovery from mechanical strain cannot be ruled out.
10. The terms cell envelope and cell wall should be explicitly described in the introduction as the text depends heavily on usage of these terms. Does envelope growth mean the growth of OM/ IM? There is no discussion on what happens to membrane growth in absence of PG insertion.

Referee #3:

Review of Oldewurtel 2022 - Cell-envelope growth of Gram-negative bacteria proceeds independently of cell-wall synthesis

This paper presents a study of the extent to which cell wall expansion is coupled to peptidoglycan synthesis in Escherichia coli. Contrary to expectations, the authors find that cell wall expansion can proceed independently of de novo cell wall synthesis. This has been shown by the same authors and others previously, but not as comprehensively as is demonstrated here. Specifically, the authors find that cell wall expansion can proceed without PG synthesis both during vegetative growth in a fixed medium, during the response to nutrient shifts and during mechanical-stress-induced differential growth. These striking findings refute the widespread belief that cell wall expansion is the direct result of cell wall synthesis, and affirm an underappreciated role of cell wall hydrolysis as the primary mechanism driving cell wall expansion. The authors further demonstrate that cell widening in response to osmotic shock is not mediated by cell-wall insertion, although given that this phenomenon is well known to happen on rapid timescales (i.e. clearly independent of PG insertion) when osmotic treatments are not ramped, this particular demonstration was perhaps unnecessary. Finally, although the results are limited to Gram-negative bacterial growth (they do not apply in, for instance, the Gram-positive bacterium Bacillus subtilis), the authors reproduce some their PG inhibition results in two other Gram-negative bacteria during vegetative growth in a fixed medium, demonstrating that this phenomenon is phylogenetically widespread within Gram-negatives.

Major Comments:

-Overall the paper is relatively comprehensive, however the one key gap is that the authors did not perform experiments on any of the D,D-endopeptidase mutants. There are three redundant endopeptidases (Singh 2012) and by repeating the antibiotic treatments on the single deletion mutants, the authors would learn whether any of these is particularly important for hydrolysis mediated growth in the absence of synthesis.

-Measuring the dose-response of the insertion-independent growth in response to the various antibiotics is missing. Similarly, it would be interesting to know if the phenomenon depends on the rate at which the antibiotic is applied. This is important because the E. coli may actually be adapting to the antibiotic on the time scale of the experiment, allowing them to perform insertion-independent growth.

Minor Comments

-The authors demonstrate that enhanced growth due to overexpression of the DD-endopeptidase MepS persists for only a short period of overexpression. The authors posit that this results from epigenetic regulation of hydrolase activity at the enzyme level. Notably, this regulation acts on a similar timescale to that of cell lysis following the arrest of MreB. The authors should discuss why this mechanism would not prevent the excessive growth and subsequent lysis they observe in cells treated with PG-synthesis inhibitors.

-The authors should provide some estimate for the timing of diffusion-mediated antibiotic propagation through the agarose pad.

-Why do cells expand their cell surface area (as measured by microscopy) by a larger fractional amount after PG synthesis inhibition than equivalently treated bulk cultures increase their OD?

- It would be interesting to provide measurements of cell width in cells overexpressing the endopeptidase MepS.

All major changes of the manuscript text are highlighted in yellow.

Referee #1:

The study by Oldewurtel et al. addresses the relationship between mass increase and cell wall homeostasis in Gram-negative bacteria. The main conclusion is that mass increase is independent of cell wall synthesis, but instead likely relies on cell wall lytic enzymes. This is a very well-designed study that addresses a crucial unknown aspect of cell envelope biology and, as a particularly exciting aspect of it, dispenses with the persistent dogma that cell wall synthesis rules all during envelope expansion. The conclusions are overall supported by the data and the appropriate controls are in place. I have only minor concerns.

Overall, while the main conclusion (PG synthesis is not required for envelope expansion) is well-supported, the evidence for autolysin involvement is indirect. Autolysin involvement logically follows from their PG inhibition experiments, but is not directly demonstrated (except for MepS overexpression, but this is in a different context than the main experimental system); conclusions regarding autolysin should thus be slightly softened (e.g., "can likely control..." in line 24

Fig. 1 would benefit from adding untreated cells as a comparison (panel 1B), as currently they only have mass growth in the presence of drugs. I think they might have these data, since they mention a slight disturbance in width homeostasis during drug treatment in the text (which presumably was compared to an untreated control). However, this is not necessary to support their main conclusion.

First, for better consistency between radioactive-labeling experiments and single-cell time-lapse microscopy, we replaced the previous experiments conducted in rich defined medium (RDM+glucose) on agarose pads (previous Fig. 1B) by the same experiments conducted in minimal medium (MM+glucose) using fluidic channels (new Fig. 1B):

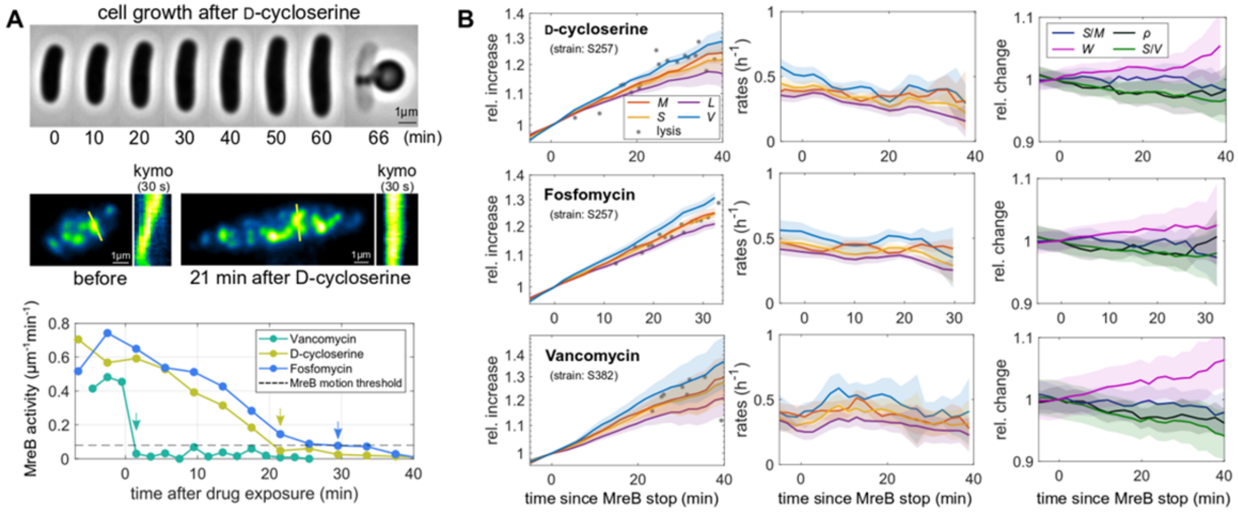

This has two advantages: a) medium and growth rates are now nearly identical to the 3H-mDAP pulse experiments (MM+glucose+lysine). b) The drug reaches the cells now nearly instantly at maximum concentration, while previously drugs reached cells through diffusion. We decided that it would still be useful to maintain the old experiments in RDM+glucose (Fig. S1D-E).

We now also conducted new 3H-mDAP-labeling experiments (Fig. S1A) with new authors Richard Wheeler, Ivo Boneca, Institut Pasteur, using the two strains (S458 for D-cycloserine, fosfomycin and S381 for vancomycin) consistent with the microscopy experiments (Fig. 1B) rather than using the imp mutant with all three drugs as done previously. Arrest of 3H-mDAP and arrest of MreB-rotation are now observed at approximately equal times (see a more detailed response to Ref. #2).

As requested, we now added control experiments for the two strains (WT and imp mutant) in a Figure S1B-C.

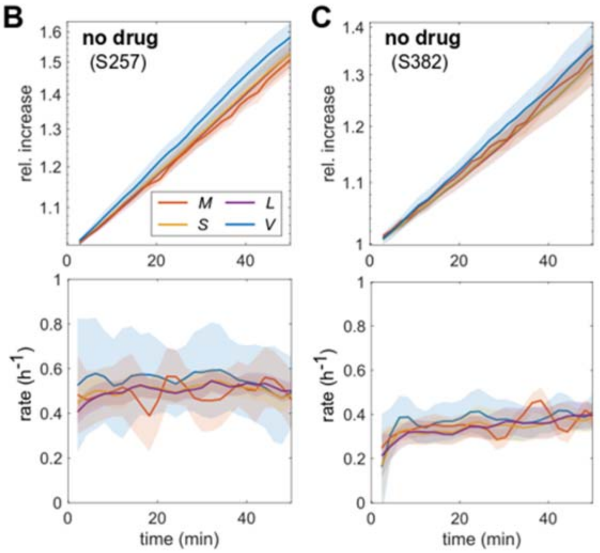

Finally, we have now replaced the measurement of average MreB speed by the measurement of MreB activity. The latter is equal to the sum of speeds of MreB filaments per cell area (and is measured by the length of MreB tracks per area per observation time). The measure reduces the noise floor while showing the same timings of MreB-motion arrest as the previous analysis.

Other, very minor comments:

- Line 24: "Therefore" should be deleted - cell change in response to mechanical stress does not logically allow for the conclusion of the involvement of autolsysin. Perhaps replace with "overall, our data suggest..."; if the statement about autolysins includes the data mentioned earlier in the abstract, rather than just mechanical stress as implied by "therefore", it makes sense

We indeed meant to refer to everything said before the statement on cell-wall-cleaving enzymes. We thus replaced “Therefore, ...” by “Overall, we conclude that ...”. We think that our observations merit a firm conclusion rather than a mere suggestion.

- Line 34/35 - there is no evidence for this (though it is logical and reasonable to think so), so perhaps weaken to "are likely co-ordinated". Their own and other labs' data actually suggest that autolysin activity and at least aPBPs are not necessarily co-ordinated in a strict sense.

We indeed did not mean to imply coordination ‘in a strict sense’. We thus followed the referee’s suggestion.

- Line 65 - "demonstrating" is a bit strong, perhaps change to "consistent with"

We agree with the referee and have changed the statement accordingly.

- Line 85: severely reduced or stopped? (though this is a minor rhetoric distinction) The figure suggests the latter.

We agree that the wording of ‘severely reduced’ was confusing, as persistent MreB rotation is clearly arrested at the times for which we previously used the term ‘severely reduced’. This is now better reflected by measuring MreB activity rather than MreB average speed (see response to the first point above). We therefore now consistently use ‘MreB-motion arrest’.

- Line 103 needs a citation, e.g. PMID: 25884840

We agree and included the citation.

- Remove extraneous sentence in line 120/121

We apologize but we could not find an extraneous sentence in line 120/21.

- Line 122 needs a citation, e.g. PMID: 23062283,
and https://doi.org/10.1111/mmi.12059

We agree and included the two citations.

- Line 139 - is this concentration/duration of treatment sufficient to stop MreB motion?

In the revised manuscript we now made sure to analyze changes of curvature (Fig. 3C,E) only from times where cell-wall synthesis is fully arrested according to independent MreB movies conducted on agarose pads with the same growth medium and containing D-cycloserine, shown in the new Movie EV14.

Because the analysis is analogous to the analysis of MreB motion for Figs. 1 and S1, we do not present the quantitative analysis of MreB rotation in the manuscript but show it here for completeness:

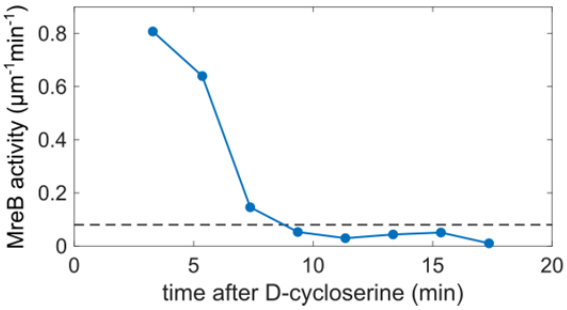

Additionally, we decided to remove the straightening experiment in the presence of fosfomycin (while leaving the straightening experiment with D-cycloserine), because 3H-mDAP insertion continues slightly longer than MreB rotation in minimal medium, leaving open the possibility that fosfomycin-treated straightening cells in RDM+glu might also continue cell-wall insertion at a low rate after arrest of MreB motion.

The new Fig. 3 is now shown in the following. It also includes a clear indication of the time of MreB-motion stop in a timeline under the snapshots.

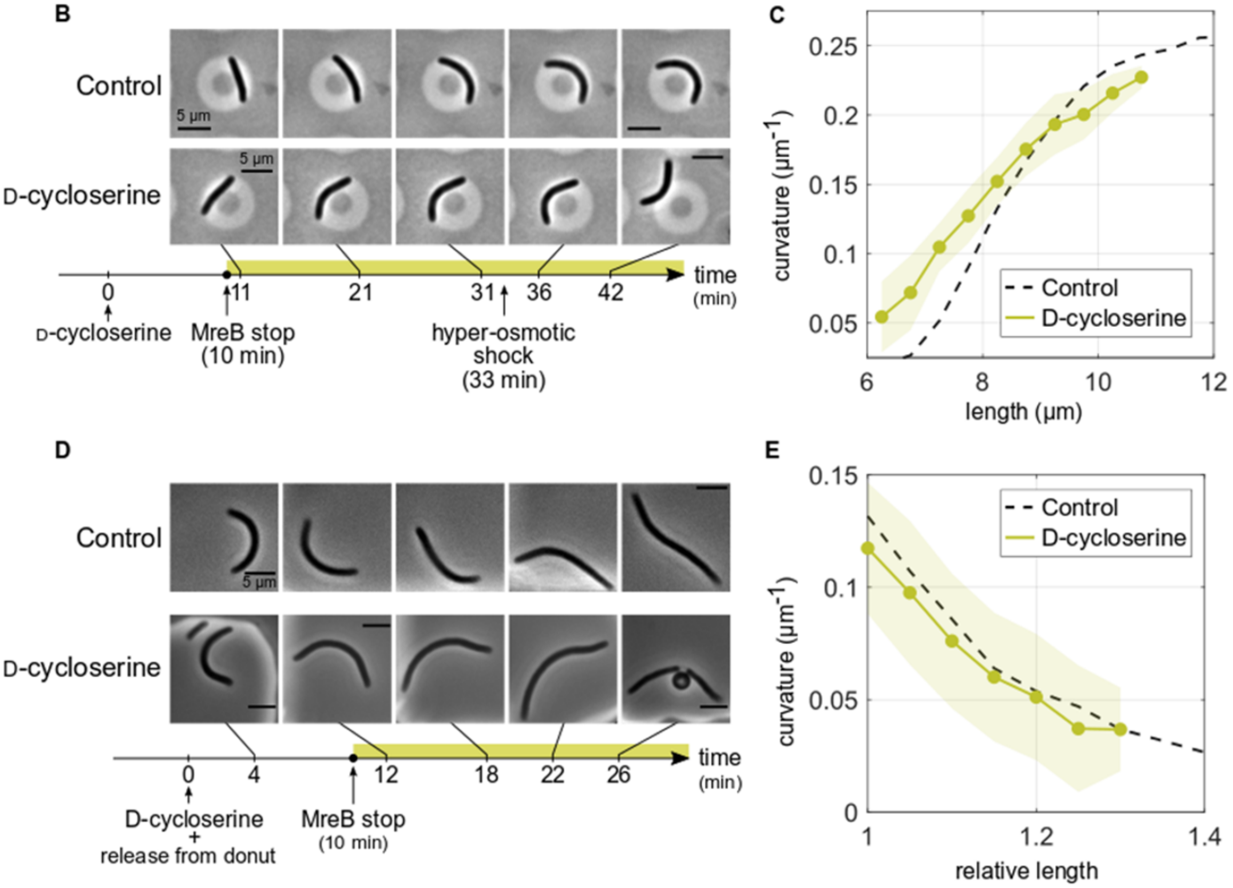

New caption of Fig. 3: *B-C: Cell bending: B: Time-lapses of bending filamentous cells (S290) in donut microchambers (3% agarose; RDM+glu) and grown without (top) or with (bottom) D-cycloserine (1 mM) as a function of time since D-cycloserine treatment, starting >10 min after D-cycloserine treatment (the time of MreB-motion stop, Movie EV14). D-cycloserine-treated cells were osmotically shocked (NaCl) after 33 min of treatment, which releases a fraction of cells, demonstrating differential envelope growth during bending (see Fig. S3A for more examples). C: Change of cell centerline curvature as a function of cell length between 10 min after start of D-cycloserine treatment and release of cells through osmotic shock (see Fig. S3B for cell length).*

D-E: Cell straightening. D: Time-lapses of straightening filamentous cells (S290) previously bent in donuts: control (top) and D-cycloserine treated cells (bottom) as a function of time since simultaneous release from donuts and beginning of drug treatment. E: Change of centerline curvature as a function of relative length from 10 min after D-cycloserine treatment (time of MreB-motion stop).

- Line 167/68 is a bit confusing, since "restoring pressure" implies an increase, while their cells actually are prevented from reducing turgor back to normal levels. Maybe replace with something like "restoring normal turgor" or similar?

We agree with the referee and have added the word ‘normal’.

- Line 285 - add more information on mass measurements. I know they refer to a previous paper, but it is easier on the reader if this procedure is explained again here.

We added the following explanation in the method part:

Mass measurements were conducted by spatial light interference microscopy (SLIM) and simulation-based image analysis as previously described in detail (13). In brief, for every phase image, we obtained 6 phase-contrast images, each taken with a different phase delay between scattered and unscattered light (differing by π/2). Those then allow calculation of the phase image through a simple algebraic relationship. From those images, the integrated phase is obtained by summing up all positive pixel values. The integrated phase is corrected for optical artifacts of the microscope by comparing the integrated phase signal with the corresponding signal from computational image simulations. The latter are obtained for every real image, based on cell contour and on the parameters of the microscope. For details see the supplementary material of (13).

Referee #2:

In this manuscript, the authors put forth an interesting hypothesis which challenges a long-held notion in bacterial physiology - that PG synthesis, or more specifically new PG insertion, is critical for cell expansion. Using extensive microscopy, authors study the rates of cell size, volume and surface increase upon inhibition of cell wall synthesis by targeting either PG precursor synthesis (fosfomycin, cycloserine) or PG polymerisation (vancomycin). Cessation of PG synthesis is verified by 3H-mDAP incorporation and reduction in MreB motion. They observe that cells continue to grow and elongate after inhibition of MreB motion, however the growth is transient and eventually cells lyse. Furthermore, such cell envelope expansion is perturbed synchronously with perturbation of biomass growth, mechanical strain and osmotic downshock.

Interestingly, the authors report that envelope growth is proportional to D,D-endopeptidase activity which raises the possibility of PG incorporation and hydrolysis being decoupled. The experiments are designed meticulously to study minute physical details of cell expansion on a short timescale.
However, the conclusions drawn here are overreaching, and the presented evidence is quite preliminary and does not sufficiently invalidate the need for PG insertion for cell envelope expansion. Several additional experiments are required to conclusively say that envelope growth does not require new PG insertion. It appears that cell envelope expansion, if truly independent of PG synthesis, is a transient event that is not physiologically sustainable as cells lyse shortly after PG synthesis inhibition.

We thank the referee for his/her careful assessment of our work.

We also fully agree with the referee that envelope growth in the absence of synthesis is a transient, physiologically not sustainable process. Nevertheless, during the short observation window between synthesis arrest and lysis we can clearly conclude that cells control the rate of envelope growth without any input from cell-wall insertion. The fact that synthesis arrest leads to lysis (and is thus not sustainable) is stated in multiple places in the manuscript. For example, we wrote in the abstract: '*[...] we found that both global and local rates of envelope growth of Escherichia coli remain nearly unperturbed upon arrest of cell-wall insertion – up to the point of sudden cell lysis.'*, and at the end of the introduction we wrote *'While cell-wall insertion remains essential for cell-width regulation, cell division, and cell-wall integrity, cell-wall cleaving autolysins control envelope growth independently of peptidoglycan insertion.'* To be very clear, we added another sentence after the first paragraph of the Discussion, writing: *While cell-wall insertion is not rate-limiting for both global and local rates of envelope growth, it is required for the maintenance of cell-wall integrity, as demonstrated by rapid cell lysis after drug treatments (Fig. 1).*

Specific points are listed below -

1. The most crucial aspect of this study to be considered is - confirming whether there is a complete inhibition of PG incorporation. The evidence presented here has two caveats. Firstly, it is unclear from the description whether the point at which the growth

monitoring starts (indicated as time since MreB stop in Fig. 1B) is the point of complete stoppage or significant reduction in MreB motion (described as 'severely reduced within 5-15 min depending on the drug' in line 85). At what time point in the movies S1-3 was growth monitoring initiated? Further, cessation of MreB motion is not indicative of absence of PG polymerisation, as aPBPs are known to work independently of the Rod complex (Cho 2016) and may still be sufficiently functional for PG incorporation here. Along the same lines, line 221 states 'Here, we found that the activity of aPBPs is not responsible for differential cell-envelope growth' - direct evidence for this has not been presented. Secondly, the point at which 3H-mDAP incorporation stops following drug treatment appears to be longer than the point at which MreB motion stops, which is where cell growth monitoring begins. Eg: It takes ~22 mins for mDAP incorporation to stop following fosfomycin treatment, although growth rate monitoring begins at ~15 mins post treatment (Figs 1A, 1B, S1A). In addition, the 3H-mDAP incorporation assay is performed in broth culture, while growth rate is monitored on agarose pad where diffusion time for the drug may lead to a further lag, because of which the time required for complete inhibition of PG incorporation may be underestimated here.

We thank the referee for the critical evaluation of our results. Before addressing the different points one by one, we would like to apologize for the confusion caused by the discrepancies between MreB-motion arrest and arrest of 3H-mDAP insertion. Those differences are likely predominantly caused by the use of two different growth media for the two experiments (RDM+glucose for microscopy, but MM+glucose for mDAP insertion). Cells previously studied in RDM+glu grew faster. They therefore arrested MreB motion faster than cells grown in MM. For consistency between microscopy and 3H-mDAP experiments, we now conducted the following experiments and analyses:

a) We conducted microscopy in MM+glucose (rather than rich medium) and used fluidic channels (rather than agar pads). The latter allowed us to change media quickly (within seconds) (Fig. 1B):

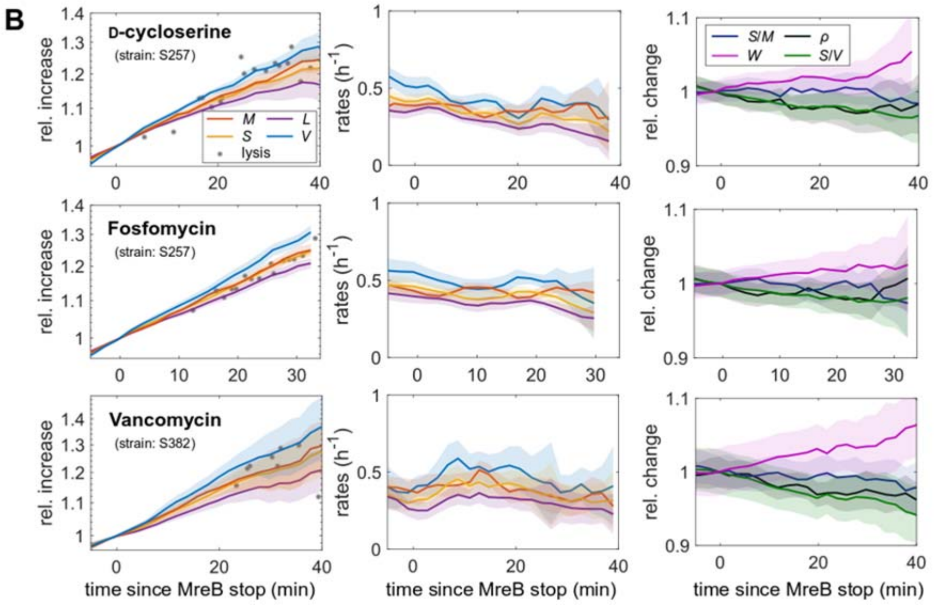

Times of MreB-motion stop are now also clearly indicated in the corresponding Fig. 1A (see point ‘e’ below and explanation below).

b) We also conducted control experiments for the same strains in the same medium (Fig. S1B-C) that show equal growth rates without drug treatment:

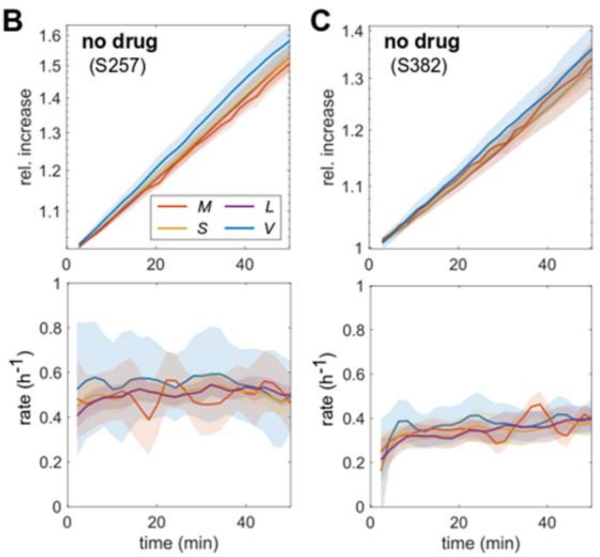

c) We repeated MreB-rotation measurements in the same MM+glucose and adjusted the analysis of MreB rotation to better reflect the arrest of persistent MreB motion (Fig. 1A):

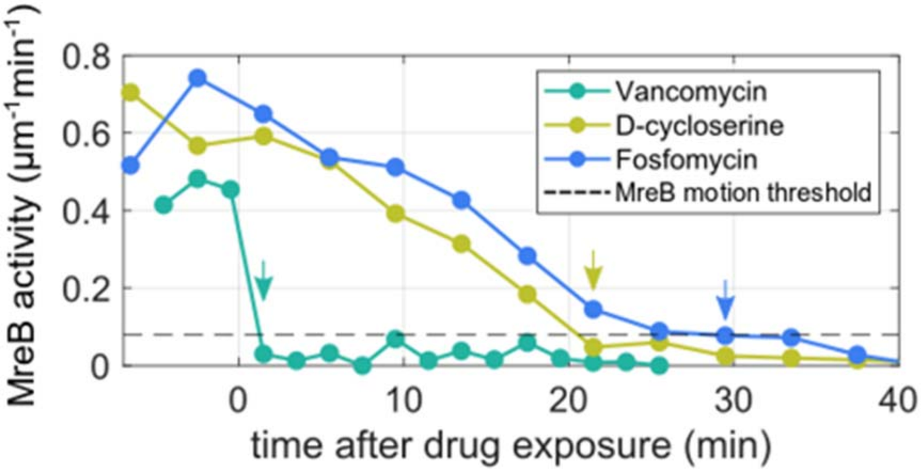

Here, we replaced the measurement of average speed by MreB activity, which is the spatial density of MreB filaments multiplied by their average speed. MreB activity increases linearly with both speed and density, thus reflecting the overall rate of MreB-based cell-wall insertion. The previous average speed showed a plateau value of about 20% of the maximum even at long times due to residual jittery motion of MreB filaments, even though cell-wall insertion is fully arrested according to 3H-mDAP insertion, possibly due to random interactions between MreB and its interaction partners. MreB activity displays a much reduced noise floor if compared to average speed, reflecting the absence of persistent motion at long times (Movies EV1-3). Note that the timing of MreB-motion arrest (indicated

by arrows) is not affected by the choice of the analysis (vancomycin: <2 min; D-cycloserine: 21 min; fosfomycin: 30 min).

d) We repeated 3H-mDAP experiments (Fig. S1A) with new authors Richard Wheeler, Ivo Boneca, Institut Pasteur, using the two strains (S458 for D-cycloserine, fosfomycin and S381 for vancomycin) consistently with the microscopy experiments (Fig. 1B) rather than using the imp mutant with all three drugs as done previously:

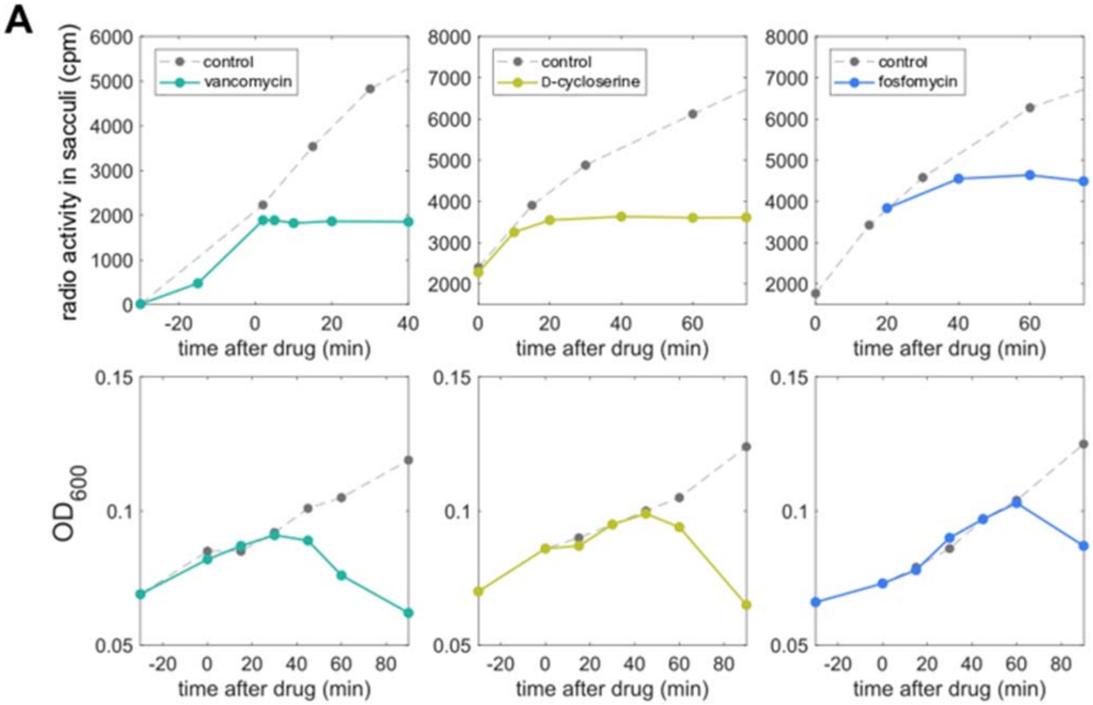

Insertion arrest is now observed at: vancomycin: <2 min; D-cycloserine: 10-20 min; fosfomycin: 20-40 min, consistent with our previous results. The uncertainty is due to the limited time resolution of the experiment. Assuming a sudden arrest of 3H-mDAP insertion as previously, arrest times are likely close to 15 min (D-cycloserine) and 30 min (fosfomcycin).

3H-mDAP and MreB-rotation measurements are thus consistent. Only fosfomycin shows a slightly longer (but strongly reduced) insertion after the time of MreB-rotation arrest according to 3H-mDAP, possibly due to residual class-A PBP activity. We still prefer to use the time of MreB-motion stop as a reference time in Fig. 1B, rather than the time of 3H-mDAP-insertion arrest, because of the slightly different growth conditions: microscopy was conducted in fluidic channels while 3H-mDAP insertion was measured in shaking liquid culture. However, our conclusions remain valid if we base the estimated times even if we use the upper time limits of 3H-mDAP-insertion arrest times (2, 20, 40 min): The latter correspond to residual surface growth of 27%, 23%, and 16% according to Fig. 1B for vancomycin, D-cycloserine, and fosfomycin.

e) We decided that it would still be useful to maintain the experiments conducted in RDM+glucose as part of the supporting figure S1. For those, we also included

steady-state growth rates for surface and mass from control experiments in the two strains used (S257 and S382).

Finally, since times of MreB-motion stop and cell-wall-synthesis arrest according to mDAP insertion are nearly identical in minimal medium, we used MreB-motion stop as a proxy for the time of cell-wall-insertion arrest also in other growth media, notably in LB and RDM+glucose, where mDAP-experiments cannot be conducted. We added a short explanation to the text when we first do so (overexpression of MepS during D-cycloserine treatment). Specifically, we write: *[...] At this point, MreB rotation was stopped in LB medium (Movie EV13) used in this experiment. Since the arrest of MreB motion is coincident with the arrest of cell-wall insertion in minimal medium (Figs. 1A, S1A), cell-wall insertion is likely also completely stopped after MreB-motion stop in LB medium. We thus use MreB-rotation arrest as a proxy for the time of cell-wall-insertion arrest in this and subsequent experiments.*

To respond to the concrete points raised by the referee:

It is unclear from the description whether the point at which the growth monitoring starts (indicated as time since MreB stop in Fig. 1B) is the point of complete stoppage or significant reduction in MreB motion (described as 'severely reduced within 5-15 min depending on the drug' in line 85

We agree that the wording of ‘severely reduced’ was confusing, as persistent MreB rotation is clearly arrested at the times for which we previously used the term ‘severely reduced’ (see also our explanation about measuring MreB activity above). We therefore now consistently use ‘MreB-motion arrest’ or ‘MreB-motion stop’.

At what time point in the movies S1-3 was growth monitoring initiated?

Growth in Fig. 1B is shown as a function of time since MreB-motion stop. The times of MreB-motion stop after drug addition are obtained from our MreB-activity analysis (Fig. 1A), in agreement with visual inspection of movies EV1-3, and in agreement with 3H-mDAP labeling (Fig. S1A). This information is now more clearly added to the figure caption of Fig. 1. We write: *MreB motion stopped 2 min (vancomycin), 21 min (D-cycloserine) or 30 min (fosfomycin) after drug addition, as indicated by arrows (see Movies EV1-3).* The information is also added to the movie caption for Movies EV1-3.

... cessation of MreB motion is not indicative of absence of PG polymerisation, as aPBPs are known to work independently of the Rod complex (Cho 2016) and may still be sufficiently functional for PG incorporation here.

We totally agree. This is the reason why we compared MreB-rotation arrest with the time of 3H-mDAP insertion in minimal medium. MreB-motion stop is coincident with the arrest of 3H-mDAP. Only for fosfomycin do we see a slightly longer 3H-mDAP incorporation time (at highly reduced rate), which is indeed possibly due to aPBP activity.

[...] line 221 states 'Here, we found that the activity of aPBPs is not responsible for differential cell-envelope growth' - direct evidence for this has not been presented.

Since surface growth continues after complete cessation of 3H-mDAP insertion, we can conclude that none of the cell-wall-synthesis machineries is responsible for envelope growth. We are referring to aPBPs specifically, because we mentioned them in the preceding sentence as a previously hypothesized regulator of cell bending and straightening. To avoid confusion, we now write: '*Here, we found that **cell-wall synthesis, including aPBP activity**, is not responsible for differential cell-envelope growth*'

Secondly, the point at which 3H-mDAP incorporation stops following drug treatment appears to be longer than the point at which MreB motion stops.

Times of MreB-motion arrest and 3H-mDAP insertion arrest now agree fully for vancomycin and D-cycloserine. Only fosfomycin shows a slightly longer insertion duration as discussed above.

In addition, the 3H-mDAP incorporation assay is performed in broth culture, while growth rate is monitored on agarose pad where diffusion time for the drug may lead to a further lag, because of which the time required for complete inhibition of PG incorporation may be underestimated here.

This is the reason why we switched to conducting growth experiments in fluidic channels.

2. Fig. 1A, movie S1 shows that cell lysis directly coincides with the point of complete cessation of MreB motion following addition of D-cycloserine (~30 min). This further brings into question the duration for which cell envelope expansion proceeds after PG incorporation has completely stopped. This event appears to be transient and not physiologically sustainable as cells do not survive for long. Is the lysis due to turgor, or due to strict requirement of PG insertion for growth? If cell envelope growth is truly independent of PG incorporation, will the cells remain viable in hyper osmotic media where turgor will not lead to lysis?

The previous Movie S1 (now replaced by a movie of MreB motion in minimal medium for the reasons pointed out above) showed that persistent MreB motion stops some time between the movie recorded at 8.5 min and the movie recorded at 16 min, in agreement with the quantitative analysis, i.e., well before lysis at 30 min. As indicated above, we do observe residual jittery (non-persistent) motion of MreB even at long times after drug treatment for all three drugs and in both rich medium (former Movies S1-3) and minimal medium (new Movies EV1-3). However, this motion cannot possibly reflect cell-wall insertion, as cell-wall insertion is clearly abolished at long times according to 3H-mDAP labeling experiments in minimal medium. This is thus also the reason why we switched our MreB analysis to MreB activity as described in detail in response to point 1. To illustrate the abolishment of persistent MreB motion after drug treatment, we now also included a new Movie EV7, for which we recorded MreB-GFP throughout 25 min of

growth after vancomycin treatment in RDM+glu with an increased time interval of 20 s. There, the continued cell elongation despite rapid and complete stop of persistent MreB rotation is clearly visible.

Growth without cell-wall insertion is indeed transient. Continued growth requires cell-wall insertion, likely because cell-wall pores get unsustainably large. As shown in previous work (Vigouroux et al. *eLife* 2020), cells also likely require repair enzymes that fix defects that build up during growth.

To stabilize cell shape in hyperosmotic conditions would be a great idea. However, while we indeed observed that cells grown in LB+500 mM NaCl and treated with D-cycloserine grow slightly longer than in low-osmolarity medium (Fig. 1B, S1B), they still lyse within about 0.5-0.7 mass-doubling times. Thus, while it will be interesting to explore other osmotic conditions in the future, we restrict the investigation to low-osmolarity media and shorter timescales in this study. Those experiments still allow us to conclude that cell-wall synthesis has no rate-limiting effect for cell-envelope growth on the timescale of about half a mass-doubling time.

3. Line 24 'cell-wall-cleaving enzymes can control envelope growth independently of synthesis' and Fig 2B, C - the experiment showing increase in cell size and volume upon overexpression of MepS does not include drug treatment for inhibition of PG incorporation. Therefore there is no direct evidence to state that the action of MepS is independent of PG synthesis.

We agree with the referee that inhibition of cell-wall insertion is pertinent for our conclusion. We therefore tested if the transient increase of elongation by MepS overexpression is independent of PG synthesis by performing MepS overexpression 15 min after placing cells on an agarose pad containing D-cycloserine (new Fig. S2D). Since these experiments were conducted in strain b183 (*ΔmepS mreB-msfGFP pBAD30-mepS*) in LB medium, we now also made sure to demonstrate that MreB motion is clearly arrested after 15 min of D-cycloserine treatment in this strain and growth medium (Movie EV13). We observed a burst of equal magnitude as in the absence of D-cycloserine, demonstrating that the burst is indeed independent of cell-wall insertion. However, we observed rapid lysis after the burst, in agreement with the requirement of cell-wall insertion for the repair of cell-wall defects, which likely accumulate upon MepS overexpression. The new Fig. S2D:

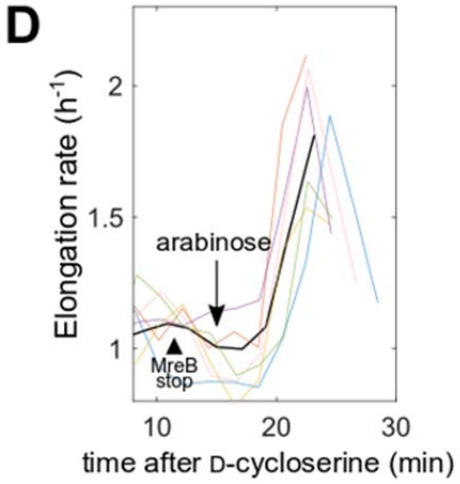

__Figure S2D caption:__ Single-cell elongation rate during MepS overexpression of strain b183 (ΔmepS mreB-msfGFP pBAD30-mepS) as a function of time after placing cells on agarose pad containing D-cycloserine (1 mM) (thin lines: single cells; thick black line: average) shows equal burst as MepS induction in non-drug-treated cells (Fig. S2B). MepS was induced 15 minutes after D-cycloserine treatment (0.2% arabinose as a droplet on top of agarose pad), well after observation of MreB-motion stop in a parallel experiment without MepS induction (11 min after drug treatment) (Movie EV13).

As a result of the large number of new experiments on MepS described here and in response to the following points, we have significantly changed the section 'Envelope growth responds transiently to variations of endopeptidase levels.'

4. Line 128 'elongation rate and surface rate increase only transiently (during 5-10 min), despite long-term enzyme overexpression, suggesting that envelope growth is likely not controlled through endopeptidase levels but through the regulation of activity at the single-enzyme level.'; Fig 2A; and lines 213-216 in discussion - was MepS expression checked over time? MepS half-life known to be very low and tightly regulated and may be reflected in the growth rate. A novel self-regulatory mechanism for MepS regulation, which has not been predicted by structural studies, need not be invoked based on this observation.

We checked MepS expression 15 min and 30 min after inducer (arabinose) addition (new Fig. S2A) by Western blot, demonstrating that MepS stays highly overexpressed throughout the experiment. Thus, rapid protein turnover is not responsible for the rapid reduction of elongation rate:

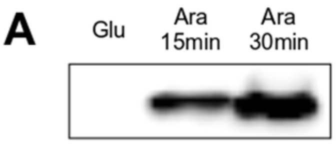

__Figure S2A caption__: MepS protein levels in the membrane fraction during growth of strain b42 (ΔmepS pBAD30-mepS) in LB+glu (0.2%) and after washing + arabinose induction (0.2%; t = 15 min and 30 min after induction) us-ing Western blot.

5. In similar lines, if one has to invoke the role of endopeptidases in insertion-independent envelope growth, then these experiments need to be repeated in appropriate mutants (lacking MepS or any other).

We observed growth in single-gene deletion mutans for *mepS*, *mepM*, and *mepH* in the presence of 1 mM D-cycloserine (Fig. S2E). All three mutants increase their surface area at non-perturbed rate until lysis. We now write in the main text:

Finally, we also tested whether cell elongation in the absence of cell-wall synthesis required a specific endopeptidase, among the redundantly essential MepM, MepH, and MepS. We thus tested the ability of the corresponding single-gene deletion mutants to continue cell elongation after D-cycloserine treatment (Fig. S2E). Single-gene deletion mutants for these endopeptidases continued to elongate after D-cyloserine treatment (Fig. S2E), well after MreB motion had arrested, as tested in the mepS mutant (around 10 min, Movie EV13), and we did not observe any differences between drug-treated and untreated cells. Thus, none of the three hydrolases is individually responsible for elongation in the absence of cell-wall insertion.

The results are shown in Fig. S2E:

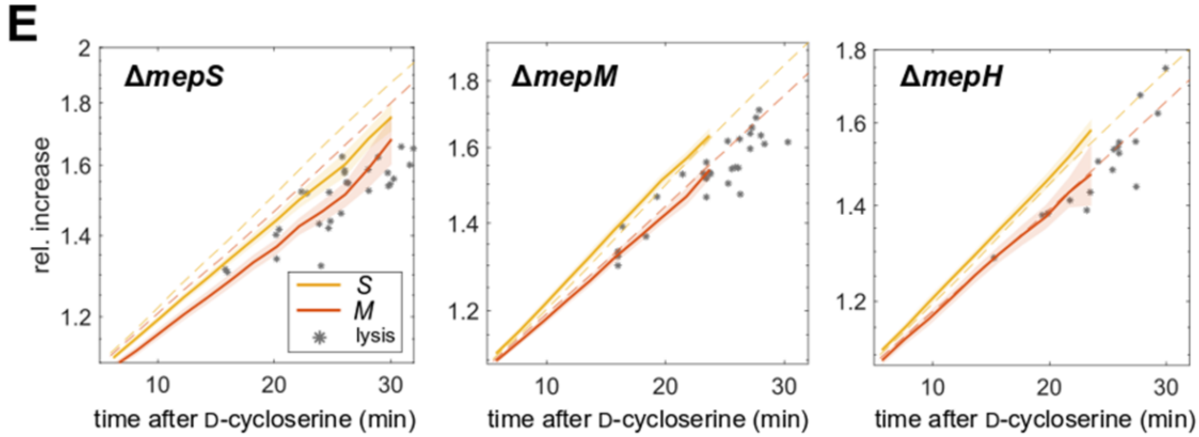

__Figure S2E caption:__ Single-cell growth of endopeptidase mutants EL54 (ΔmepS), KS81 (ΔmepM) and KS82 (ΔmepH) grown in LB+D-cycloserine (1 mM) as a function of time after placing cells on agarose pad containing the drug. Relative increase of surface and mass. Gray asterisks indicate single-cell mass at lysis and dashed lines indicate averages in the absence of drug.

Due to the large redundancy of DD-endopeptidases, this result is possibly expected. In the future, we thus plan to study growth in mutants that lack multiple endopeptidases. However, given the focus of this paper on the role of cell-wall synthesis for growth, and given this study would require a lot of time and work, such a study goes beyond the scope of this paper.

6. Line 96: The authors suggest that "cell-wall cleaving autolysins must be responsible for extending the area of the cell wall, independent of cell-wall insertion", since E. coli PG is mostly single-layered, how such process will allow PG extension without affecting PG integrity should be discussed. This is a point of particular interest as it can further explain the process of PG remodeling and re-structuring without new PG incorporation. Will endopeptidase deficient mutants fail in such process?

We do not mean to say that cell-wall insertion is dispensable for long-term viability. Instead, it is essential to prevent lysis (and to maintain shape on long time scales). We now introduced a sentence after the first paragraph of the Discussion, writing:

While cell-wall insertion is not rate-limiting for both global and local rates of envelope growth, it is required for the maintenance of cell-wall integrity, as demonstrated by rapid cell lysis after drug treatments (Fig. 1). As already stated in response to the introductory point of Ref. #2, we mention the importance of cell-wall insertion to prevent cell lysis in multiple places of the manuscript.

We have an additional paragraph of the Discussion dedicated to the role of cell-wall insertion for cell shape.

7. Line 119: Endopeptidase other than MepS can alter envelope growth? Because of the importance of the observation, as a control experiment MepS catalytic mutant should be used. More MepS may also titrate NlpI leading to reregulation of several hydrolases/ synthases (Banzhaf, 2020).

We did not test the ability of other endopeptidases to alter envelope growth, given their large degree of redundancy. However, we are planning to do similar experiments with other endopeptidases in the future. Since this article is focused on the role of cell-wall insertion for envelope growth, we think that experiments with further endopeptidases would go beyond the scope of this paper.

However, we agree that control experiments with an inactive MepS mutant are important. We thus expressed the MepS C68A mutant and demonstrate the absence of a burst in the new Fig. 2B, together with the empty-vector control. Additionally, we tested and demonstrated the dose-dependent response of MepS to varying levels of inducer in the same figure:

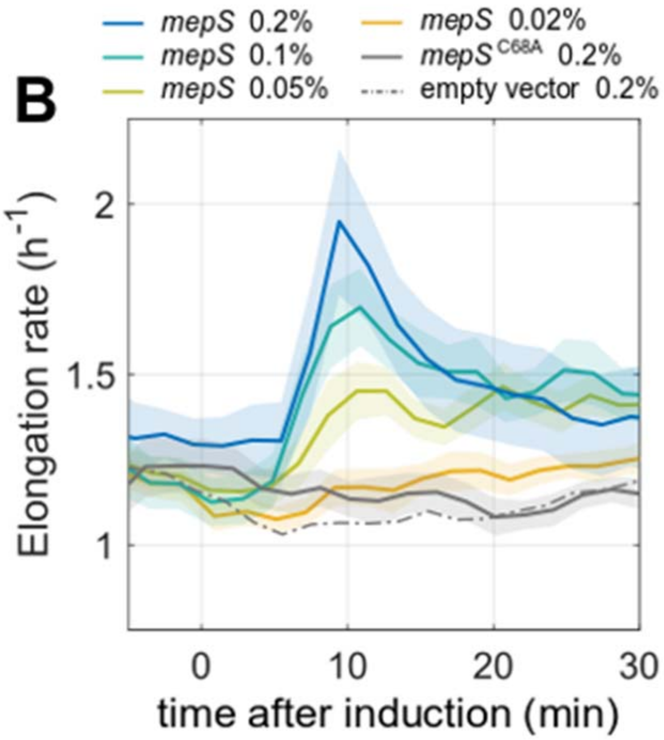

Caption Figure 2B: *Elongation rates upon MepS overexpression as in A but for different induction levels (0.02-0.2% arabinose), and including empty-vector control (b55) and inactive mutant (S606 ΔmepS/pBAD30-mepS^{C68A}).*

We also confirmed expression of the MepS mutant and the dose-dependent expression of the wild-type protein by Western blot (measured 20 min after induction) in a new Fig. S2C:

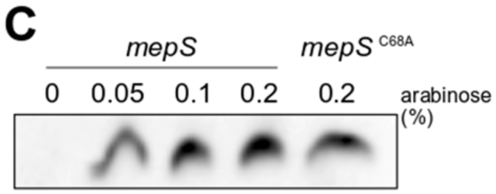

Figure caption Fig. S2C:

MepS protein levels in the membrane fraction during growth of strains b42 (ΔmepS pBAD30-mepS) or S606 (ΔmepS pBAD30-mepS^{C68A}) in LB measured 20 min after induction (0.05-0.2% arabinose) using Western blot.

We agree that the inactivation of other endopeptidases could also contribute to the downregulation of envelope growth. We have thus included a sentence in the Results section reading: *While the rapid inactivation of other autolysins might also contribute to the reduction of envelope growth, the fact that MepS is strongly overexpressed in our experiments, suggests that MepS cannot simply be constitutively active after the elongation burst. Thus, envelope growth is likely not controlled through endo-peptidase levels but through the regulation of activity at the single-enzyme level.*

As a result of the large number of new experiments on MepS we have significantly changed the section 'Envelope growth responds transiently to variations of endopeptidase levels.'

8. Lines 76-78: Logic of using SulA overexpression to inhibit FtsZ is not clear. None of the antibiotics used in this study mediate septation-induced lysis (Ref 26 talks about beta-lactam induced cell lysis).

We agree with the referee that none of the antibiotic used is specifically known for leading to lysis during division. Our major reason for expressing SulA is to facilitate image analysis and thus to allow long-term imaging of single cells during time-lapse microscopy. We thus removed the argument about septation-induced cell lysis from the text. However, in preliminary experiments with dividing cells we observed early lysis at mid-cell if treated with D-cycloserine.

9. Fig 3D - the duration of recovery (~20 mins) for both D-cycloserine and fosfomycin appears to be within the window between drug addition and cessation of mDAP incorporation. Therefore PG insertion playing a role in recovery from mechanical strain cannot be ruled out.

Different from experiments in Fig. 1A-B, bending and straightening experiments were done in RDM+glucose. As explained in response to point 1, we now use MreB activity as a proxy for the arrest of cell-wall insertion, since the time of MreB-motion stop and cell-wall-insertion arrest are coincident in minimal medium (for D-cycloserine and vancomycin). MreB motion is arrested 10 min after D-cycloserine exposure in RDM+glu (see the new Movie EV14). The time of MreB motion stop was obtained by analysis of MreB activity. Because the analysis is analogous to the analysis of MreB motion for Figs. 1 and S1, we do not present the quantitative analysis of MreB rotation in the manuscript but show it here for completeness:

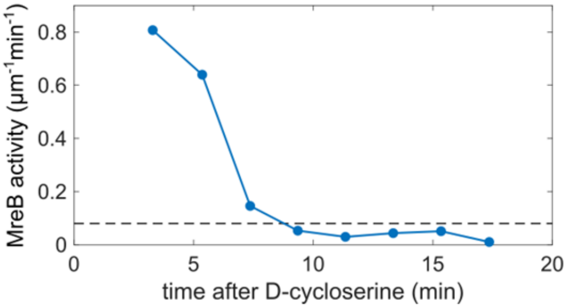

Additionally, we decided to remove the straightening experiment in the presence of fosfomycin (while leaving the straightening experiment with D-cycloserine), because 3H-mDAP insertion continues slightly longer than MreB rotation in minimal medium, leaving open the possibility that fosfomycin-treated straightening cells in RDM+glu might also continue cell-wall insertion at a low rate after arrest of MreB motion.

The new Fig. 3 is now shown in the following. It also includes a clear indication of the time of MreB-motion stop in a timeline under the snapshots.

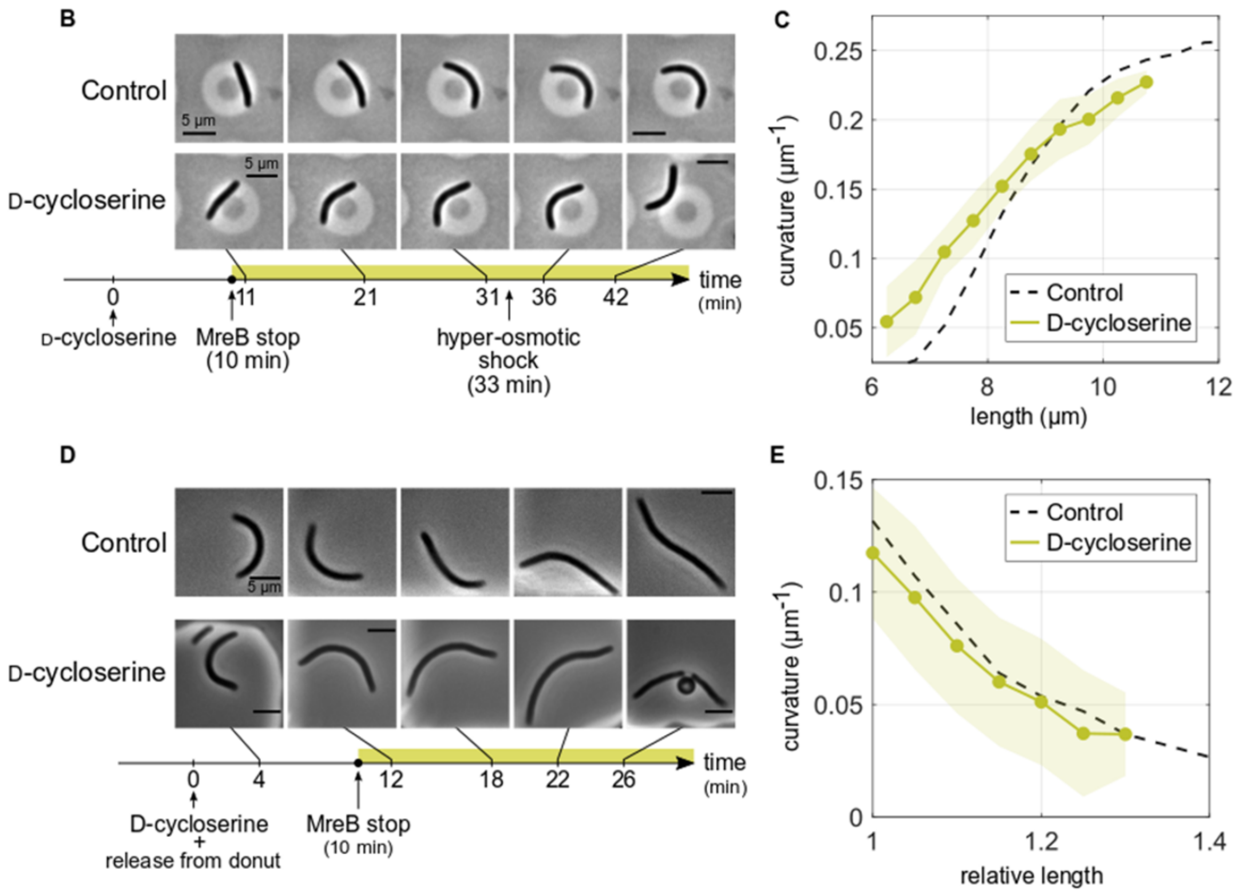

New caption of Fig. 3: *B-C: Cell bending: B: Time-lapses of bending filamentous cells (S290) in donut microchambers (3% agarose; RDM+glu) and grown without (top) or with (bottom) D-cycloserine (1 mM) as a function of time since D-cycloserine treatment, starting >10 min after D-cycloserine treatment (the time of MreB-motion stop, Movie EV14). D-cycloserine-treated cells were osmotically shocked (NaCl) after 33 min of treatment, which releases a fraction of cells, demonstrating differential envelope growth during bending (see Fig. S3A for more examples). C: Change of cell centerline curvature as a function of cell length between 10 min after start of D-cycloserine treatment and release of cells through osmotic shock (see Fig. S3B for cell length).*

D-E: Cell straightening. D: Time-lapses of straightening filamentous cells (S290) previously bent in donuts: control (top) and D-cycloserine treated cells (bottom) as a function of time since simultaneous release from donuts and beginning of drug treatment. E: Change of centerline curvature as a function of relative length from 10 min after D-cycloserine treatment (time of MreB-motion stop).

10. The terms cell envelope and cell wall should be explicitly described in the introduction as the text depends heavily on usage of these terms. Does envelope growth mean the growth of OM/ IM? There is no discussion on what happens to membrane growth in absence of PG insertion.

The cell envelope comprises both membranes and the cell wall. We now added this information to the introduction, writing: *In Gram-negative bacteria, such as Escherichia*

coli, the cell wall is a thin layer with a thickness of a few nanometers (5, 6), which is sandwiched between the two other envelope components, the cytoplasmic membrane and the outer membrane.

When we say ‘envelope growth’ we mean the increase of envelope surface area. This is in analogy to ‘size growth’ referring to the increase of cell volume, and ‘mass growth’ referring to the increase of cell mass. All these quantities are defined independently of the abundance of envelope/volume/mass components. We now define ‘cell-envelope growth’ in the introduction, when writing *Accordingly, cell-wall insertion might be rate-limiting for the global rate of cell-envelope growth (1, 2, 7, 8),* ***that is the global increase of cell-surface area****, and for the local rates of cell-envelope growth, [...].*

We now included a new paragraph in the Discussion, where we discuss the coordination of growth of the outer membrane, the cell wall, and the cytoplasmic membrane:

Cell-wall insertion does likely not affect the growth of any envelope component

The observation that cell-wall-synthesis inhibition does not affect surface-area growth implies that the outer membrane continues to grow in surface area at an unperturbed rate independently of cell-wall insertion. Whether its structural or mechanical properties are affected, remains to be studied in the future. Additionally, we reason that the inner membrane must also continue to grow, because cells don’t show any sign of plasmolysis. The latter should be visible if the cytoplasmic volume did not increase while total volume increases by 30% (Fig. 1B). A mild reduction in cytoplasmic-membrane growth would be harder to detect and can thus not be fully ruled out, but given the fact that cells continue to grow at unperturbed rates in surface area and mass after drug treatment suggests to us that cell physiology, and thus also cell envelope geometry and membrane integrity, are likely not severely perturbed.

Referee #3:

Review of Oldewurtel 2022 - Cell-envelope growth of Gram-negative bacteria proceeds independently of cell-wall synthesis

This paper presents a study of the extent to which cell wall expansion is coupled to peptidoglycan synthesis in Escherichia coli. Contrary to expectations, the authors find that cell wall expansion can proceed independently of de novo cell wall synthesis. This has been shown by the same authors and others previously, but not as comprehensively as is demonstrated here. Specifically, the authors find that cell wall expansion can proceed without PG synthesis both during vegetative growth in a fixed medium, during the response to nutrient shifts and during mechanical-stress-induced differential growth. These striking findings refute the widespread belief that cell wall expansion is the direct result of cell wall synthesis, and affirm an underappreciated role of cell wall hydrolysis as the primary mechanism driving cell wall expansion. The authors further demonstrate that cell widening in response to osmotic shock is not mediated by cell-wall insertion, although given that this phenomenon is well known to happen on rapid timescales (i.e. clearly independent of PG insertion) when osmotic treatments are not ramped, this particular demonstration was perhaps unnecessary. Finally, although the results are limited to Gram-negative bacterial growth (they do not apply in, for instance, the Gram-positive bacterium Bacillus subtilis), the authors reproduce some their PG inhibition results in two other Gram-negative bacteria during vegetative growth in a fixed medium, demonstrating that this phenomenon is phylogenetically widespread within Gram-negatives.

We thank the referee for appreciating the important points of this paper.

Regarding the referee’s point about cell widening upon hypo-osmotic ramp: In our experiments, cells increase their width by about 8% within 20 min. This increase of width cannot be explained by elastic stretching alone, since a sudden hypoosmotic shock of similar magnitude (520 mOsm) only causes a width increase of 2.5% (see Fig. S17 of Oldewurtel et al. PNAS 2021). Similar values of width changes were measured by the lab of Teuta Pilizota (Edinburgh) for an osmotic shock of 460 mOsm using a mutant that lacks the major mechanosensitive channels MscS and MscL (raw data underlying Fig. 2 of Buda et al. *PNAS* 2016 according to personal communication).

We now explain this logic in the results section, based on our previous work, writing at the beginning of the paragraph: *We previously demonstrated that E. coli cells change their cell width plastically and in a dose-dependent manner in response to changes of cytoplasmic osmotic pressure (13). To that end, we conducted diffusion-limited NaCl-based hypo-osmotic ramps similar to the one illustrated in Fig. 4A. Here, an agarose pad of low salt concentration is added on top of an agarose pad of high concentration, which leads to the continuous decreases of medium osmolality at the position the coverslip by about 500 mOsm over the course of 20 min (Fig. 4B). Since medium osmolality keeps on changing during this time frame, cytoplasmic turgor is likely elevated throughout the ramp (13). During the 500 mOsm ramp of Fig. 4, control cells (labeled as ‘Hypo ramp’ in Fig. 4C) widen by about 8%, in agreement with previous*

observations (13). The 8% increase of width cannot be explained by elastic stretching alone but requires plastic cell widening, since cells exposed to a sudden hypo-osmotic shock of similar magnitude (520 mOsm) show instantaneous elastic widening of only about 2.5% (13).

Additionally, we now indicate more clearly the fold-change of cell width in the new experiment (in the presence of D-cycloserine) together with the magnitude of the hypo-osmotic ramp.

Major Comments:

-Overall the paper is relatively comprehensive, however the one key gap is that the authors did not perform experiments on any of the D,D-endopeptidase mutants. There are three redundant endopeptidases (Singh 2012) and by repeating the antibiotic treatments on the single deletion mutants, the authors would learn whether any of these is particularly important for hydrolysis mediated growth in the absence of synthesis.

We now included experiments with single-gene deletion mutants for *mepS*, *mepM*, and *mepH* in the presence of 1 mM D-cycloserine (Fig. S2E). All the mutants increase their surface area at non-perturbed rate until lysis. We now write in the main text:

Finally, we also tested whether cell elongation in the absence of cell-wall synthesis required a specific endopeptidase, among the redundantly essential MepM, MepH, and MepS. We thus tested the ability of the corresponding single-gene deletion mutants to continue cell elongation after D-cycloserine treatment (Fig. S2E). Single-gene deletion mutants for these endopeptidases continued to elongate after D-cyloserine treatment (Fig. S2E), well after MreB motion had arrested, as tested in the mepS mutant (around 10 min, Movie EV13), and we did not observe any differences between drug-treated and untreated cells. Thus, none of the three hydrolases is individually responsible for elongation in the absence of cell-wall insertion.

The results are shown in Fig. S2E:

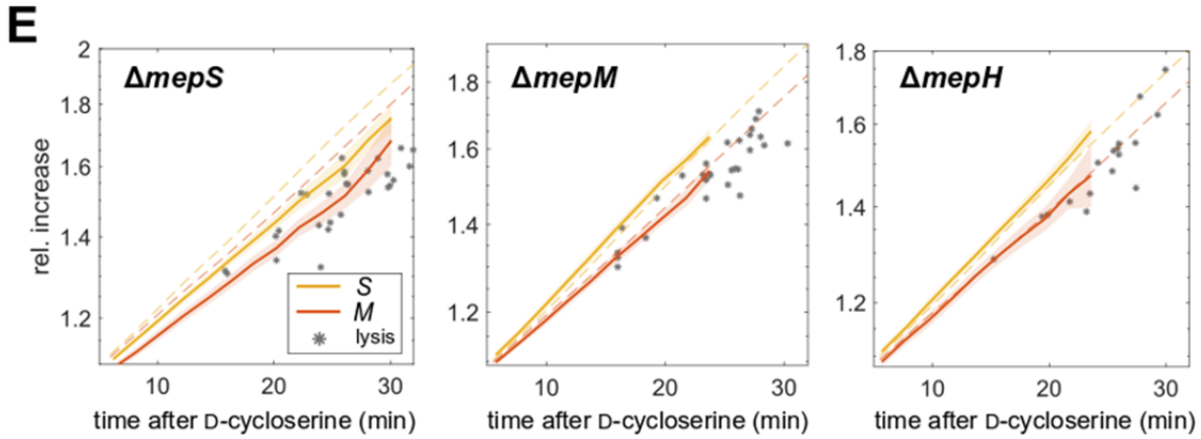

Figure S2E caption: *Single-cell growth of endopeptidase mutants EL54 (ΔmepS), KS81 (ΔmepM) and KS82 (ΔmepH) grown in LB+D-cycloserine (1 mM) as a function of time after placing cells on agarose pad containing the drug. Relative increase of surface and mass. Gray asterisks indicate single-cell mass at lysis and dashed lines indicate averages in the absence of*

drug.

-Measuring the dose-response of the insertion-independent growth in response to the various antibiotics is missing. Similarly, it would be interesting to know if the phenomenon depends on the rate at which the antibiotic is applied. This is important because the E. coli may actually be adapting to the antibiotic on the time scale of the experiment, allowing them to perform insertion-independent growth.

In our experiments we treat cells with drugs at high enough concentrations (vancomycin 100 µg/mL, D-cycloserine 1 mM, fosfoymcin 500 µg/mL) to fully arrest cell wall insertion and as fast as possible. The concentrations are saturating and at lease 10 times above MIC according to the following growth curves in MM+glu liquid medium:

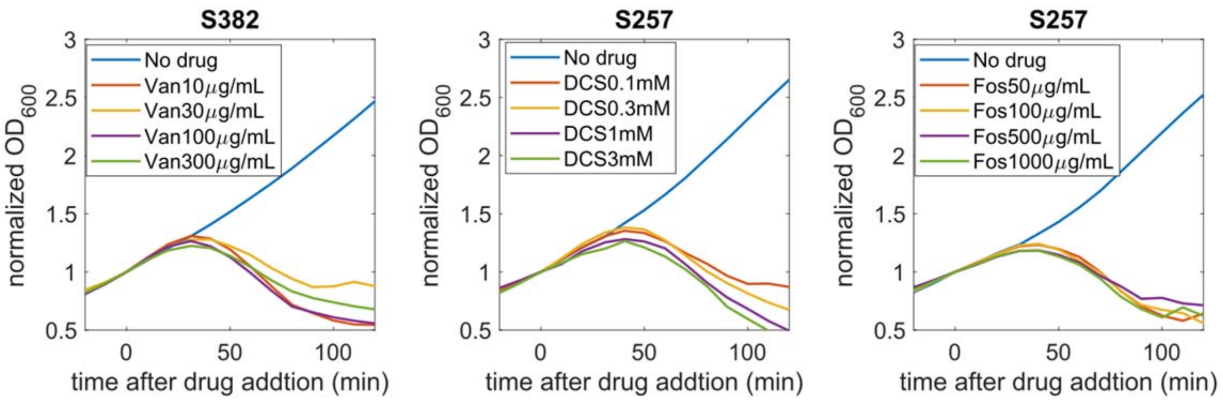

We did not fully understand the referee's argument. Possibly, the referee speculates that cells might show a reduction of growth rate at low drug concentration but not at high concentration, since they then have more time to prepare for the stress.

We do not expect any effect of the drugs at lower concentrations. However, we note that the same 'non-effect' is observed with different applications of the same drug (pad vs channel), which gives the cell more or less time to 'adjust' to the antibiotic stress. On this note, in response to Referees #1 and #2 we replaced experiments in Fig. 1 by the same experiments conducted in flow chambers (using minimal medium). For details see the extensive response to Ref. #2. For example, if added to agarose pads, it takes about 5 min for vancomycin to reach 1% of its final concentration by diffusion, and about 9 min for 10% of final concentration, while the drug arrives essentially instantaneously in the flow chambers.

Additionally, the response to antibiotics is identical for three different types of drugs that show a fast (vancomycin), intermediate (d-cyc), or slow (fosfomycin) reduction of cell-wall insertion according to both MreB rotation (Fig. 1A, S1B) and 3H-mDAP insertion (Fig. S1A). For example, fosfomycin treatment leads to a steady, continuous reduction of MreB activity over the course of about 30 min in minimal medium (Fig. 1A) and over the course of about 15 min in rich medium (Fig. S1D). Yet, the rate of surface-area growth does not significantly change during these time courses (Fig. 1B, Fig. S1E). We now included a sentence pointing out this observation, writing *We also note that both D-cycloserine and fosfomycin treatment leads to a steady, continuous reduction of MreB*

activity over the course of about 20 min or 30 min prior to arrest, respectively. Yet, surface-area growth does not change during or after these time frames (Fig. 1B).

Finally, from the liquid-growth curves above it is apparent that growth continues up to the point of cell lysis, at an unperturbed rate, independently of whether drug concentration is high or low (tested over a 30-fold range).

We thus decided not to conduct further microscopy experiments with even lower concentrations of drugs.

Minor Comments

-The authors demonstrate that enhanced growth due to overexpression of the DD-endopeptidase MepS persists for only a short period of overexpression. The authors posit that this results from epigenetic regulation of hydrolase activity at the enzyme level. Notably, this regulation acts on a similar timescale to that of cell lysis following the arrest of MreB. The authors should discuss why this mechanism would not prevent the excessive growth and subsequent lysis they observe in cells treated with PG-synthesis inhibitors.

The cell apparently continues to expand its cell wall if MepS is not overexpressed (or after the MepS burst is finished (see new Fig. 2A)). Why cells do not use this type of mechanism to arrest hydrolytic activity to coordinate with cell wall synthesis is a great question and not at all clear to us. This is different from the behavior in *B. subtilis*, where we previously reported that cell-wall expansion slows down a lot after arrest of cell-wall insertion.

-The authors should provide some estimate for the timing of diffusion-mediated antibiotic propagation through the agarose pad.

Based on one-dimensional diffusion model, cells experience 10% of the final concentration of vancomycin within approximately 9 min. We added this information in the method part. To be more accurate about the time of drug exposure to cells for our central experiment (Fig. 1A-B), we now conducted those experiments in flow chambers (Fig. 1A-B), while moving the diffusion-based drug treatments conducted previously to Fig. S1D-E.

-Why do cells expand their cell surface area (as measured by microscopy) by a larger fractional amount after PG synthesis inhibition than equivalently treated bulk cultures increase their OD?

Previously the conditions of bulk experiment and single-cell microscopy were different in terms of the growth medium and strains. To be consistent, we now used the same medium (MM+glu) and the strains with the same background for both bulk and microscopy experiments. We observed that after cell wall synthesis stop, cells grow by a similar amount in microscopy (15-30%) and bulk culture (10-20%). We mentioned it as

follows in the main text: *Consistent with microscopy analyses, drug-treated batch cultures continue to grow without perturbation by approximately 10-20% (Fig. S1A).*

Thus, single-cell growth proceeds for a slightly shorter relative amount in minimal medium than in rich medium, for a currently unknown reason.

- It would be interesting to provide measurements of cell width in cells overexpressing the endopeptidase MepS.

We showed the relative change of cell width in Fig. 2A (previous Fig. 2B) and single cell traces in Fig. S2B (previous Fig S2). We also provide the absolute value in Table S1.

1st Revision - Editorial Decision 5th May 2023

Dear Sven,

Thank you for submitting a revised version of your manuscript. Your study has now been seen by all of the original referees, who find that their previous concerns have been addressed and now recommend publication of the manuscript. There remain only a couple of minor editorial points that have to be addressed before I can extend formal acceptance of the manuscript:

1. Please address the minor textual points by referees #1 and #2.
2. Please rename "Bibliography" section into "References".
3. Please move the Supplementary Methods to the main manuscript text.
4. Please update the nomenclature for Appendix Tables S1-S2 and Appendix Figures S1-S4 in the manuscript text and in the Appendix file.
5. Papers published in The EMBO Journal are accompanied online by a 'Synopsis' to enhance discoverability of the manuscript. It consists of A) a short (1-2 sentences) summary of the findings and their significance, B) 3-4 bullet points highlighting key results and C) a synopsis image that is 550x300-600 pixels large (width x height, jpeg or png format). You can either show a model or key data in the synopsis image. Please note that the image size is rather small and that text needs to be readable at the final size. Please send us this information together with the revised manuscript.

Please let me know if you have any further questions regarding any of these points. You can use the link below to upload the revised files.

Thank you again for giving us the chance to consider your manuscript for The EMBO Journal. I look forward to receiving the final version.

With best wishes,

Ieva

Ieva Gailite, PhD
Senior Scientific Editor
The EMBO Journal
Meyerhofstrasse 1
D-69117 Heidelberg
Tel: +4962218891309
i.gailite@embojournal.org

Further information is available in our Guide For Authors: https://www.embopress.org/page/journal/14602075/authorguide

We realize that it is difficult to revise to a specific deadline. In the interest of protecting the conceptual advance provided by the work, we recommend a revision within 3 months (3rd Aug 2023). Please discuss the revision progress ahead of this time with the editor if you require more time to complete the revisions.

Referee #1:

All my concerns have been sufficiently addressed. I would encourage the authors to correct two further minor issues:

1. Line 55 (and possibly elsewhere): Should be Miguel De Pedro, not Petro.
2. lines 117 vs 122 poses an apparent contradiction: Elongation "continues at an unperturbed rate" in line 117, but "is slightly reduced" in line 122. This could probably be fixed by "elongation continues at nearly unperturbed rate" in line 117

Referee #2:

Revised manuscript reads well and I have no major concerns.
A minor point include:
Lines 301- "transcriptional" needs to be removed. MepS is modulated at the enzyme stability level.

Referee #3:

My major and minor comments were adequately addressed. This rich study will be a valuable addition to the bacterial cell biology field.

The authors have addressed all minor editorial requests.

3rd Revision - Editorial Decision **16th May 2023**

Dear Sven,

Thank you for addressing the final editorial points. I am now pleased to inform you that your manuscript has been accepted for publication. The provisional DOI of your manuscript is DOI: 10.15252/embj.2022112168

Before we forward your manuscript to our publishers, I would like to propose a couple of minor changes in the article title, synopsis and abstract to increase their accessibility to our more general readership. I have also written a short blurb that will accompany the title of your manuscript in our online table of contents. Please take a look at the text below and in the attached manuscript text file and let me know if any corrections are needed.

Title:
Cell envelope growth of Gram-negative bacteria proceeds independently of cell wall synthesis

Blurb:
E. coli and S. enterica can increase their surface area and respond to mechanical forces by adapting their shape without inserting new cell wall material

Synopsis:
Bacterial cell wall insertion is often thought to drive and control cell envelope growth. Here, findings in Gram-negative bacteria show that cell wall insertion is essential for envelope integrity, but has no regulatory role for the global rate of surface area growth and for the response of cell shape to mechanical forces.
- Upon complete arrest of cell wall insertion, E. coli and S. enterica serovar Typhimurium cells continue to grow their cell envelope proportionally to biomass growth.
- Strong overexpression of the cell wall-cleaving hydrolase MepS transiently increases the rate of E. coli envelope growth, revealing tight regulation at longer time scales.
- E. coli cells are able to sense and respond to bending forces independently of cell wall insertion.
- E. coli cells inhibited for cell wall insertion are able to increase their cell diameter with increasing cytoplasmic pressure.

Please note that it is EMBO Journal policy for the transcript of the editorial process (containing referee reports and your response letter) to be published as an online supplement to each paper. If you do NOT want this, you will need to inform the Editorial Office via email immediately. More information is available here:
https://www.embopress.org/page/journal/14602075/authorguide#transparentprocess

Your manuscript will be processed for publication in the journal by EMBO Press. Manuscripts in the PDF and electronic editions of The EMBO Journal will be copy edited, and you will be provided with page proofs prior to publication. Please note that supplementary information is not included in the proofs.

You will be contacted by Wiley Author Services to complete licensing and payment information. The required 'Page Charges Authorization Form' is available here: https://www.embopress.org/pb-assets/embo-site/tej_apc.pdf - please download and complete the form and return to embopressproduction@wiley.com

EMBO Press participates in many Publish and Read agreements that allow authors to publish Open Access with reduced/no publication charges. Check your eligibility: https://authorservices.wiley.com/author-resources/Journal-Authors/open-access/affiliation-policies-payments/index.html

Should you be planning a Press Release on your article, please get in contact with embojournal@wiley.com as early as possible, in order to coordinate publication and release dates.

If you have any questions, please do not hesitate to call or email the Editorial Office. Thank you for this contribution to The EMBO Journal and congratulations on a successful publication!

Best wishes,

Ieva

Ieva Gailite, PhD
Senior Scientific Editor
The EMBO Journal
Meyerhofstrasse 1

D-69117 Heidelberg
Tel: +4962218891309
i.gailite@embojournal.org

Corresponding Author Name: Sven van Teeffelen	
Journal Submitted to: The EMBO Journal	
Manuscript Number: EMBOJ-2022-112168	

USEFUL LINKS FOR COMPLETING THIS FORM
The EMBO Journal - Author Guidelines
EMBO Reports - Author Guidelines
Molecular Systems Biology - Author Guidelines
EMBO Molecular Medicine - Author Guidelines

Reporting Checklist for Life Science Articles (updated January
This checklist is adapted from Materials Design Analysis Reporting (MDAR) Checklist for Authors. MDAR establishes a minimum set of requirements in transparent reporting in the life sciences (see Statement of Task: 10.31222/osf.io/9sm4x). Please follow the journal's guidelines in preparing your manuscript.
Please note that a copy of this checklist will be published alongside your article.

Abridged guidelines for figures
1. Data
The data shown in figures should satisfy the following conditions:
- the data were obtained and processed according to the field's best practice and are presented to reflect the results of the experiments in an accurate and unbiased manner.
- ideally, figure panels should include only measurements that are directly comparable to each other and obtained with the same assay.
- plots include clearly labeled error bars for independent experiments and sample sizes. Unless justified, error bars should not be shown for technical
- if n<5, the individual data points from each experiment should be plotted. Any statistical test employed should be justified.
- Source Data should be included to report the data underlying figures according to the guidelines set out in the authorship guidelines on Data

2. Captions
Each figure caption should contain the following information, for each panel where they are relevant:
- a specification of the experimental system investigated (eg cell line, species name).
- the assay(s) and method(s) used to carry out the reported observations and measurements.
- an explicit mention of the biological and chemical entity(ies) that are being measured.
- an explicit mention of the biological and chemical entity(ies) that are altered/varied/perturbed in a controlled manner.
- the exact sample size (n) for each experimental group/condition, given as a number, not a range;
- a description of the sample collection allowing the reader to understand whether the samples represent technical or biological replicates (including how many animals, litters, cultures, etc.).
- a statement of how many times the experiment shown was independently replicated in the laboratory.
- definitions of statistical methods and measures:
  - common tests, such as t-test (please specify whether paired vs. unpaired), simple χ2 tests, Wilcoxon and Mann-Whitney tests, can be unambiguously identified by name only, but more complex techniques should be described in the methods section;
  - are tests one-sided or two-sided?
  - are there adjustments for multiple comparisons?
  - exact statistical test results, e.g., P values = x but not P values < x;
  - definition of 'center values' as median or average;
  - definition of error bars as s.d. or s.e.m.

Please complete ALL of the questions below.
Select "Not Applicable" only when the requested information is not relevant for your study.

Materials

Newly Created Materials	Information included in the manuscript?	In which section is the information available? (Reagents and Tools Table, Materials and Methods, Figures, Data Availability Section)
New materials and reagents need to be available; do any restrictions apply?	Not Applicable	

Antibodies	Information included in the manuscript?	In which section is the information available? (Reagents and Tools Table, Materials and Methods, Figures, Data Availability Section)
For **antibodies** provide the following information:<br>- Commercial antibodies: RRID (if possible) or supplier name, catalogue number and or/clone number<br>- Non-commercial: RRID or citation	Yes	Materials and Methods

DNA and RNA sequences	Information included in the manuscript?	In which section is the information available? (Reagents and Tools Table, Materials and Methods, Figures, Data Availability Section)
**Short novel DNA or RNA including primers, probes**: provide the sequences.	Yes	Appendix Supplementary Methods

Cell materials	Information included in the manuscript?	In which section is the information available? (Reagents and Tools Table, Materials and Methods, Figures, Data Availability Section)
**Cell lines:** Provide species information, strain. Provide accession number in repository **OR** supplier name, catalog number, clone number, and/**OR** RRID.	Not Applicable	
**Primary cultures:** Provide species, strain, sex of origin, genetic modification status.	Not Applicable	
Report if the cell lines were recently **authenticated** (e.g., by STR profiling) and tested for mycoplasma contamination.	Not Applicable	

Experimental animals	Information included in the manuscript?	In which section is the information available? (Reagents and Tools Table, Materials and Methods, Figures, Data Availability Section)
**Laboratory animals or Model organisms:** Provide species, strain, sex, age, genetic modification status. Provide accession number in repository **OR** supplier name, catalog number, clone number, **OR** RRID.	Not Applicable	
**Animal observed in or captured from the field:** Provide species, sex, and age where possible.	Not Applicable	
Please detail **housing and husbandry conditions**.	Not Applicable	

Plants and microbes	Information included in the manuscript?	In which section is the information available? (Reagents and Tools Table, Materials and Methods, Figures, Data Availability Section)
**Plants:** provide species and strain, ecotype and cultivar where relevant, unique accession number if available, and source (including location for collected wild specimens).	Not Applicable	
**Microbes:** provide species and strain, unique accession number if available, and source.	Yes	Appendix Table S3

Human research participants	Information included in the manuscript?	In which section is the information available? (Reagents and Tools Table, Materials and Methods, Figures, Data Availability Section)
If collected and within the bounds of privacy constraints report on age, sex and gender or ethnicity for all study participants.	Not Applicable	

Core facilities	Information included in the manuscript?	In which section is the information available? (Reagents and Tools Table, Materials and Methods, Figures, Data Availability Section)
If your work benefited from core facilities, was their service mentioned in the acknowledgments section?	Not Applicable	

Design

Study protocol	Information included in the manuscript?	In which section is the information available? (Reagents and Tools Table, Materials and Methods, Figures, Data Availability Section)

If study protocol has been **pre-registered, provide DOI in the manuscript**. For clinical trials, provide the trial registration number **OR** cite DOI.	Not Applicable	
Report the **clinical trial registration number** (at ClinicalTrials.gov or equivalent), where applicable.	Not Applicable	

Laboratory protocol	Information included in the manuscript?	In which section is the information available? (Reagents and Tools Table, Materials and Methods, Figures, Data Availability Section)
Provide DOI OR other citation details if **external detailed step-by-step protocols** are available.	Not Applicable	

Experimental study design and statistics	Information included in the manuscript?	In which section is the information available? (Reagents and Tools Table, Materials and Methods, Figures, Data Availability Section)
Include a statement about **sample size** estimate even if no statistical methods were used.	Not Applicable	
Were any steps taken to minimize the effects of subjective bias when allocating animals/samples to treatment (e.g. **randomization procedure**)? If yes, have they been described?	Not Applicable	
Include a statement about **blinding** even if no blinding was done.	Not Applicable	
Describe **inclusion/exclusion criteria** if samples or animals were excluded from the analysis. Were the criteria pre-established?<br><br>If sample or data points were omitted from analysis, report if this was due to attrition or intentional exclusion and provide justification.	Not Applicable	
For every figure, are **statistical tests** justified as appropriate? Do the data meet the assumptions of the tests (e.g., normal distribution)? Describe any methods used to assess it. Is there an estimate of variation within each group of data? Is the variance similar between the groups that are being statistically compared?	Not Applicable	

Sample definition and in-laboratory replication	Information included in the manuscript?	In which section is the information available? (Reagents and Tools Table, Materials and Methods, Figures, Data Availability Section)
In the figure legends: state number of times the experiment was **replicated** in laboratory.	Yes	Figures and Appendix
In the figure legends: define whether data describe **technical or biological replicates**.	Yes	Figures and Appendix

Ethics

Ethics	Information included in the manuscript?	In which section is the information available? (Reagents and Tools Table, Materials and Methods, Figures, Data Availability Section)
Studies involving **human participants**: State details of **authority granting ethics approval** (IRB or equivalent committee(s), provide reference number for approval.	Not Applicable	
Studies involving **human participants**: Include a statement confirming that **informed consent** was obtained from all subjects and that the experiments conformed to the principles set out in the WMA Declaration of Helsinki and the Department of Health and Human Services Belmont Report.	Not Applicable	
Studies involving **human participants:** For publication of **patient photos**, include a statement confirming that consent to publish was obtained.	Not Applicable	
Studies involving experimental **animals**: State details of **authority granting ethics approva**l (IRB or equivalent committee(s), provide reference number for approval. Include a statement of compliance with ethical regulations.	Not Applicable	
Studies involving **specimen and field samples:** State if relevant **permits** obtained, provide details of authority approving study; if none were required, explain why.	Not Applicable	

Dual Use Research of Concern (DURC)	Information included in the manuscript?	In which section is the information available? (Reagents and Tools Table, Materials and Methods, Figures, Data Availability Section)
Could your study fall under dual use research restrictions? Please check biosecurity documents and list of **select agents and toxins** (CDC): https://www.selectagents.gov/sat/list.htm	Not Applicable	
If you used a select agent, is the security level of the lab appropriate and reported in the manuscript?	Not Applicable	
If a study is subject to dual use research of concern regulations, is the name of the **authority granting approval and reference number** for the regulatory approval provided in the manuscript?	Not Applicable	

Reporting

The MDAR framework recommends adoption of discipline-specific guidelines, established and endorsed through community initiatives. Journals have their own policy about requiring specific guidelines and recommendations to complement MDAR.

Adherence to community standards	Information included in the manuscript?	In which section is the information available? (Reagents and Tools Table, Materials and Methods, Figures, Data Availability Section)
State if relevant guidelines or checklists (e.g., **ICMJE, MIBBI, ARRIVE, PRISMA**) have been followed or provided.	Not Applicable	
For **tumor marker prognostic studies,** we recommend that you follow the **REMARK** reporting guidelines (see link list at top right). See author guidelines, under 'Reporting Guidelines'. Please confirm you have followed these guidelines.	Not Applicable	
For **phase II and III randomized controlled trials**, please refer to the **CONSORT** flow diagram (see link list at top right) and submit the CONSORT checklist (see link list at top right) with your submission. See author guidelines, under 'Reporting Guidelines'. Please confirm you have submitted this list.	Not Applicable	

Data Availability

Data availability	Information included in the manuscript?	In which section is the information available? (Reagents and Tools Table, Materials and Methods, Figures, Data Availability Section)
Have **primary datasets** been deposited according to the journal's guidelines (see 'Data Deposition' section) and the respective accession numbers provided in the Data Availability Section?	Yes	Data Availability Section
Were **human clinical and genomic datasets** deposited in a public access-controlled repository in accordance to ethical obligations to the patients and to the applicable consent agreement?	Not Applicable	
Are **computational models** that are central and integral to a study available without restrictions in a machine-readable form? Were the relevant accession numbers or links provided?	Not Applicable	
If publicly available data were reused, provide the respective **data citations in the reference list**.	Not Applicable	
